# Draxin from neocortical neurons controls the guidance of thalamocortical projections into the neocortex

**DOI:** 10.1038/ncomms10232

**Published:** 2015-12-14

**Authors:** Yohei Shinmyo, M. Asrafuzzaman Riyadh, Giasuddin Ahmed, Iftekhar Bin Naser, Mahmud Hossain, Hirohide Takebayashi, Hiroshi Kawasaki, Kunimasa Ohta, Hideaki Tanaka

**Affiliations:** 1 Faculty of Life Sciences, Department of Developmental Neurobiology, Kumamoto University, Kumamoto 860-8556, Japan; 2 Department of Biophysical Genetics, Graduate School of Medical Sciences, Kanazawa University, Takara-machi 13-1, Ishikawa 920–8640, Japan; 3 Division of Neurobiology and Anatomy, Graduate School of Medical and Dental Sciences, Niigata University, Niigata 951–8510, Japan; 4 Brain/Liver Interface Medicine Research Center, Kanazawa University, Ishikawa 920-8640, Japan

## Abstract

The thalamocortical tract carries sensory information to the neocortex. It has long been recognized that the neocortical pioneer axons of subplate neurons are essential for thalamocortical development. Herein we report that an axon guidance cue, draxin, is expressed in early-born neocortical neurons, including subplate neurons, and is necessary for thalamocortical development. In *draxin*^−/−^ mice, thalamocortical axons do not enter the neocortex. This phenotype is sufficiently rescued by the transgenic expression of *draxin* in neocortical neurons. Genetic interaction data suggest that draxin acts through Deleted in colorectal cancer (DCC) and Neogenin (Neo1), to regulate thalamocortical projections *in vivo*. Draxin promotes the outgrowth of thalamic axons *in vitro* and this effect is abolished in thalamic neurons from *Dcc* and *Neo1* double mutants. These results suggest that draxin from neocortical neurons controls thalamocortical projections into the neocortex, and that this effect is mediated through the DCC and Neo1 receptors.

## Abstract (web summary)

During neural development thalamocortical axons follow corticofugal projections into the neocortex. Here, using a combination of knock down and rescue experiments, the authors show that Draxin expression in neocortical cells promotes thalamic axon projections from the internal capsule.

## Graphical abstract

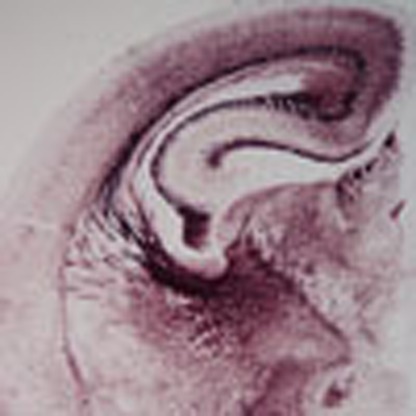

In the mammalian brain, reciprocal connections between the neocortex and thalamus formed by the corticothalamic and thalamocortical axons are critical for the relay and processing of sensory information. During development, corticothalamic and thalamocortical axons concurrently grow into the subcortical telencephalon, where they meet to form the internal capsule and continue to extend in opposite directions to reach their targets^1^. It has been known for over two decades that the guidance of thalamocortical projections is dependent on the neocortical subplate neurons, which pioneer the corticofugal pathway from the neocortex to the internal capsule^2^,^3^,^4^. Regional chemical ablation of subplate neurons leads to the disrupted thalamocortical innervation of corresponding cortical regions. Tbr1 (refs 5, 6), Coup-tf1 (ref. 7) and Fez-like^8^,^9^ transcription factor mutants provided further evidence for the importance of the subplate in thalamocortical development. Mutations in these factors lead to the defective formation of the subplate and misguidance of thalamocortical axons. Analysis of conditional mutant mice lacking corticofugal axons has shown that descending corticofugal axons are essential for guiding thalamocortical axons into the neocortex^10^. It has been recently reported that Linx, an LIG gene family transmembrane protein, mediates the interaction between corticofugal and thalamocortical axons^11^. Linx expressed on corticofugal axons is necessary for thalamocortical development, although binding partner of Linx expressed on thalamocortical axons remains unknown. Although it has been suggested that the interaction between corticofugal and thalamocortical axons is critical for the proper formation of thalamocortical projections, the molecular mechanisms underlying this interaction remain unclear.

We previously reported that a chemorepulsive axon guidance protein, draxin, which shares no significant homology with known guidance cues, is necessary for the development of spinal cord and forebrain commissures^12^. As *draxin* is expressed in the neocortical neurons of the developing brain^12^, in this study, we examined whether *draxin* is involved in establishing the reciprocal interactions of corticofugal and thalamocortical axons. We found that *draxin*^−/−^ mice had severe defects in corticofugal and thalamocortical projections. These corticofugal and thalamocortical phenotypes were rescued by the transgenic expression of *draxin* in neocortical neurons. We showed that draxin genetically interacted with Deleted in colorectal cancer (DCC) and Neogenin (Neo1). Notably, *draxin*^*+/−*^;*Dcc*^−/−^, *draxin*^*+/−*^;*Neo1*^Gt/Gt^ or *Neo1*^Gt/Gt^;*Dcc*^−/−^ mutants displayed severe corticofugal and thalamocortical projection defects, which resembled those in the *draxin*^−/−^ mice. We observed that the pathfinding errors of thalamocortical axons occurred before those of corticofugal axons in *draxin*^−/−^ mice, suggesting that draxin plays a direct role in guiding thalamocortical projections. Furthermore, we showed that draxin promotes the axonal growth of thalamic neurons *in vitro*, which was absent in thalamic neurons from *Neo1*^Gt/Gt^;*Dcc*^−/−^ embryos. These results suggest that draxin from neocortical neurons controls the guidance of thalamocortical projections and this effect of draxin is mediated through the DCC and Neo1 receptors.

## Results

### Thalamocortical phenotypes in *draxin* ^−/−^ mice

**Figure 1:**
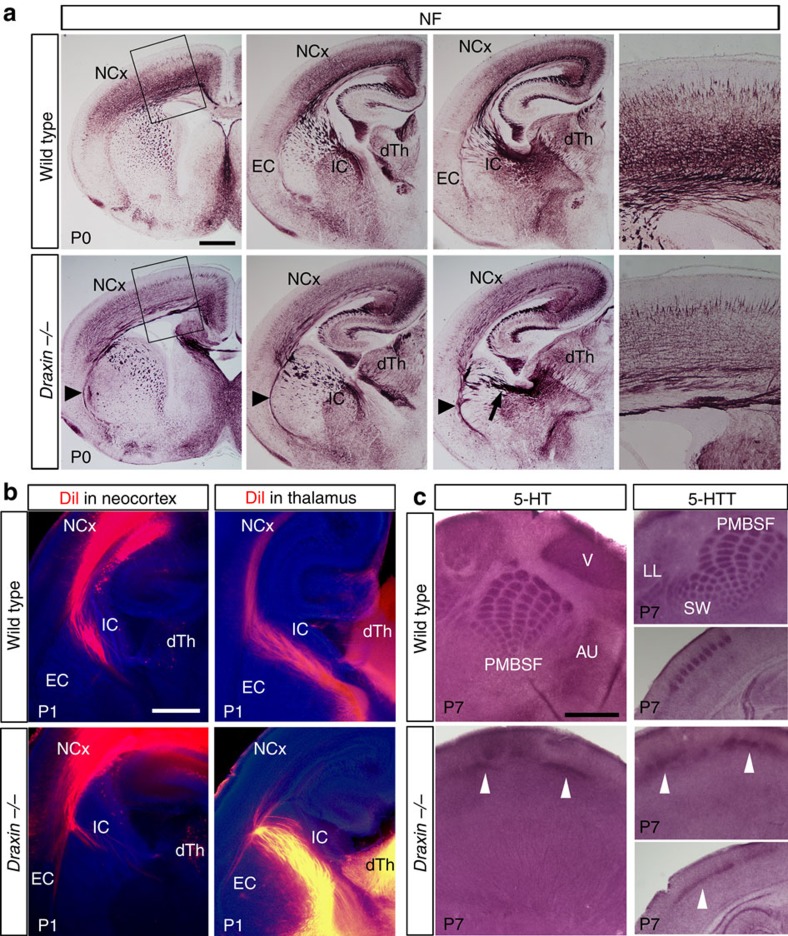
*Draxin*^−/−^ mice have severe defects in corticofugal and thalamocortical projections. (**a**) Serial coronal sections of P0 brains from wild-type and *draxin*^−/−^ mice stained with an NF antibody. The boxed areas in the left panels are enlarged in the corresponding right panels. The number of NF-positive axons was reduced in the internal capsule (arrow), whereas aberrant signals were observed along the external capsule in *draxin*^−/−^ mice (arrowheads, *n*=10 for each genotype). Scale bar, 500 μm. (**b**) Coronal sections of P1 brains from wild-type and *draxin*^−/−^ mice injected with DiI into the neocortex and dorsal thalamus. Corticofugal and thalamocortical axons exhibited pathfinding errors before entering the internal capsule and neocortex in *draxin*^−/−^ mice, respectively (*n*=12 for each genotype). Scale bar, 500 μm. (**c**) Tangential and coronal sections from the brains of wild-type and *draxin*^−/−^ mice stained with 5-HT and 5-HT transporter (5-HTT) antibodies. Arrowheads indicate a small number of thalamocortical axons entering the neocortex without any specific pattern in *draxin*^−/−^ mice (*n*=10 for each genotype). Scale bar, 100 μm. AU, primary auditory cortex; dTh, dorsal thalamus; EC, external capsule; IC, internal capsule; LL, lower lip; NCx, neocortex; PMBSF, posteromedial barrel subfield; SW, small whiskers of anterior snout; V, primary visual cortex.

To examine axon guidance defects in *draxin*^−/−^ mice, we analysed serial paraffin-embedded coronal sections of wild-type and *draxin*^−/−^ brains using immunostaining against neurofilament (NF). Corticofugal and thalamocortical axons pass through the internal capsule by forming fasciculated axon bundles in wild-type mice (Fig. 1a, upper panels). In *draxin*^−/−^ mice, the number of NF-positive axons increased in the external capsule (Fig. 1a, arrowheads), while the thickness of axon bundles reduced in the internal capsule (Fig. 1a, arrow). In addition, the density of NF-positive axons was robustly lower throughout the neocortex of *draxin*^−/−^ mice than in that of wild-type mice (Fig. 1a). To visualize corticofugal and thalamocortical axons, we performed axonal tracing experiments by injecting 1,1′-dioctadecyl-3,3,3′,3′-tetramethylindocarbocyanine perchlorate (DiI) into the neocortex and dorsal thalamus (Fig. 1b). In *draxin*^−/−^ mice, some corticofugal axons did not enter the internal capsule and instead they grew into the external capsule (Fig. 1b, left panels). Thalamocortical axons of *draxin*^−/−^ mice grew into the internal capsule but the majority of them either stalled or turned laterally towards the external capsule (Fig. 1b, right panels). We also examined the trajectories of corticofugal axons using immunostaining against TAG-1 (also known as Cntn2)^13^,^14^. Consistent with the DiI analysis, TAG-1-positive corticofugal axons entering the internal capsule were reduced in *draxin*^−/−^ mice (Supplementary Fig. 1a, arrow). In addition, we observed that some of the TAG-1-positive axons in *draxin*^−/−^ mice entered the internal capsule and reached the thalamus (Supplementary Fig. 1a, arrowhead). These results suggest that draxin is required for the normal guidance of corticofugal axons from the neocortex to the internal capsule. Furthermore, two-colour fluorescent tracing showed that in *draxin*^−/−^ mice, thalamocortical and corticofugal axons were associated in an ectopic tract within the external capsule, in addition to the internal capsule (Supplementary Fig. 1b, arrowheads). These results indicate that in *draxin*^−/−^ mice, thalamocortical and corticofugal axons followed an ectopic route through the external capsule instead of projecting to the neocortex and internal capsule, respectively. Next, we performed immunostaining against serotonin (5-HT) and the 5-HT transporter (also known as Slc6a4) to label thalamocortical axon terminals in the neocortex (Fig. 1c)^15^,^16^. We observed that their expression patterns were severely disrupted in various sensory cortices, including the somatosensory, visual and auditory cortices, of *draxin*^−/−^ mice (Fig. 1c, arrowheads), indicating that most thalamocortical axons did not innervate the neocortex properly. To examine the regional specificity of thalamocortical projection deficits in *draxin*^−/−^ mice, we analysed serial coronal sections from the entire neocortex using immunostaining against netrin-G1, which is a marker for thalamocortical axons^17^. We found that netrin-G1-positive thalamocortical axons were notably decreased throughout the neocortex of *draxin*^−/−^ mice (Supplementary Fig. 1c,d), suggesting no regional defects in the thalamocortical phenotype.

**Figure 2:**
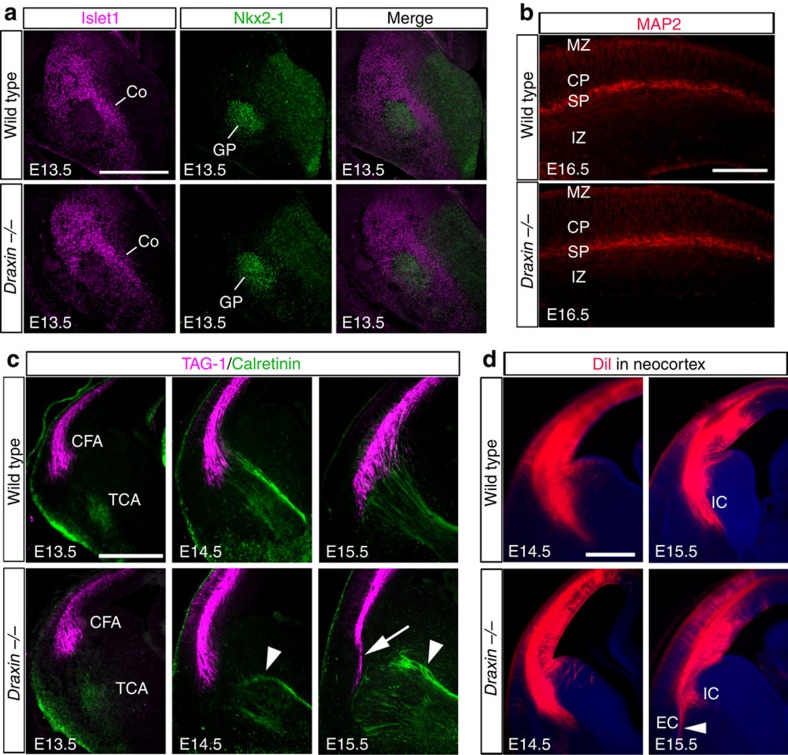
Aberrant thalamocortical projections precede aberrant corticofugal projections. (**a**) Coronal sections from E13.5 brains of wild-type and *draxin*^−/−^ mice stained with Islet1 and Nkx2-1 antibodies. The corridor and the globus pallidus cells were formed normally in *draxin*^−/−^ mice (*n*=8 for each genotype). Scale bar, 500 μm. (**b**) Coronal sections from E16.5 brains of wild-type and *draxin*^−/−^ mice stained with a MAP2 antibody. MAP2 expression was not affected in the neocortex of *draxin*^−/−^ mice (*n*=8 for each genotype). Scale bar, 300 μm. (**c**) Coronal sections from E13.5–E15.5 brains of wild-type and *draxin*^−/−^ mice stained with TAG-1 and calretinin antibodies. Arrowheads and the arrow indicate misprojected thalamocortical and corticofugal axons, respectively (*n*=8 for each genotype). Scale bar, 500 μm. (**d**) Coronal sections from E14.5 and E15.5 brains of wild-type and *draxin*^−/−^ mice with a DiI injection in the neocortex. In *draxin*^−/−^ mice, DiI-labelled axons from the neocortex correctly projected towards the internal capsule at E14.5, but some DiI-labelled axons misprojected towards the external capsule at E15.5 (arrowhead, *n*=5 for each genotype). Scale bar, 500 μm. CFA, corticofugal axons; Co, corridor cells; CP, cortical plate; GP, globus pallidus; IC, internal capsule; IZ, intermediate zone; MZ, marginal zone; SP, subplate; TCA, thalamocortical axons.

Why do corticofugal and thalamocortical axons misproject in *draxin*^−/−^ mice? The ventral telencephalon contains the corridor and globus pallidus cells, which are essential for thalamocortical axon pathfinding^18^,^19^. Corridor cells express Islet-1 and Ebf1, whereas globus pallidus cells express Nkx2-1 and Lhx6 (ref. 20). To determine whether the ventral telencephalon is defective in *draxin*^−/−^ mice, we performed double immunostaining against Islet-1 and Nkx2-1, and *in situ* hybridization using *Ebf1* and *Lhx6* probes. We observed that their expression was not affected in *draxin*^−/−^ mice (Fig. 2a and Supplementary Fig. 2a,b), suggesting that the formation of the ventral telencephalon is normal in *draxin*^−/−^ mice.

Subplate neurons play an important role in the development of subsequent corticofugal and thalamocortical axons. Therefore, we examined the formation of the subplate in *draxin*^−/−^ mice using immunostaining against MAP2, Tbr1 and chondroitin sulfate proteoglycan^6^,^21^. Expression patterns of these markers were comparable in the subplate of wild-type and *draxin*^−/−^ mice (Fig. 2b and Supplementary Fig. 2c), indicating the normal differentiation and distribution of subplate neurons. Furthermore, immunostaining for the layer-specific markers Brn2 (layers II/III, V), Ctip2 (layer V) and Foxp2 (layer VI)^22^ showed that the formation of neocortical layers was not affected in *draxin*^−/−^ mice (Supplementary Fig. 2d). We next investigated the spatiotemporal relationship between corticofugal and thalamocortical axons using double immunostaining against TAG-1 and calretinin (also known as Calb2), which are markers for neocortical^13^,^14^ and thalamic axons^23^, respectively. Axons from neocortical pioneer neurons reach the pallial–subpallial boundary (PSPB) at embryonic day 13.5 (E13.5) and lie at the lateral striatum until E15.5 (ref. 24). This waiting period enables the arrival of reciprocal thalamocortical axons at the PSPB^25^. At E13.5 and E14.5, the projection patterns of TAG-1-positive axons in *draxin*^−/−^ mice were indistinguishable from those in wild-type mice (Fig. 2c). This result indicates that neocortical pioneer axons project normally towards the internal capsule and pause in the lateral striatum of *draxin*^−/−^ mice. At E14.5, calretinin-positive thalamocortical axons progressed through the PSPB to reach the neocortex in wild-type mice (Fig. 2c). In contrast, the thalamocortical axons of *draxin*^−/−^ mice did not reach the PSPB at E14.5 and instead they misrouted towards the external capsule by E15.5 (Fig. 2c, arrowheads). At E15.5, TAG-1-positive axons did not extend into the internal capsule of *draxin*^−/−^ mice (Fig. 2c, arrow). To clearly visualize the trajectories of corticofugal axons in *draxin*^−/−^ mice, we injected DiI into the neocortex at E14.5 and E15.5. Although projection patterns from the neocortex of wild-type and *draxin*^−/−^ mice at E14.5 were similar, some DiI-labelled axons misprojected towards the external capsule at E15.5 in *draxin*^−/−^ mice (Fig. 2d, arrowhead). Thus, the pathfinding errors of thalamocortical axons precede those of corticofugal axons in *draxin*^−/−^ mice, suggesting that draxin plays a direct role in guiding thalamocortical projections from the internal capsule to the PSPB.

### Draxin expression during thalamocortical development

**Figure 3:**
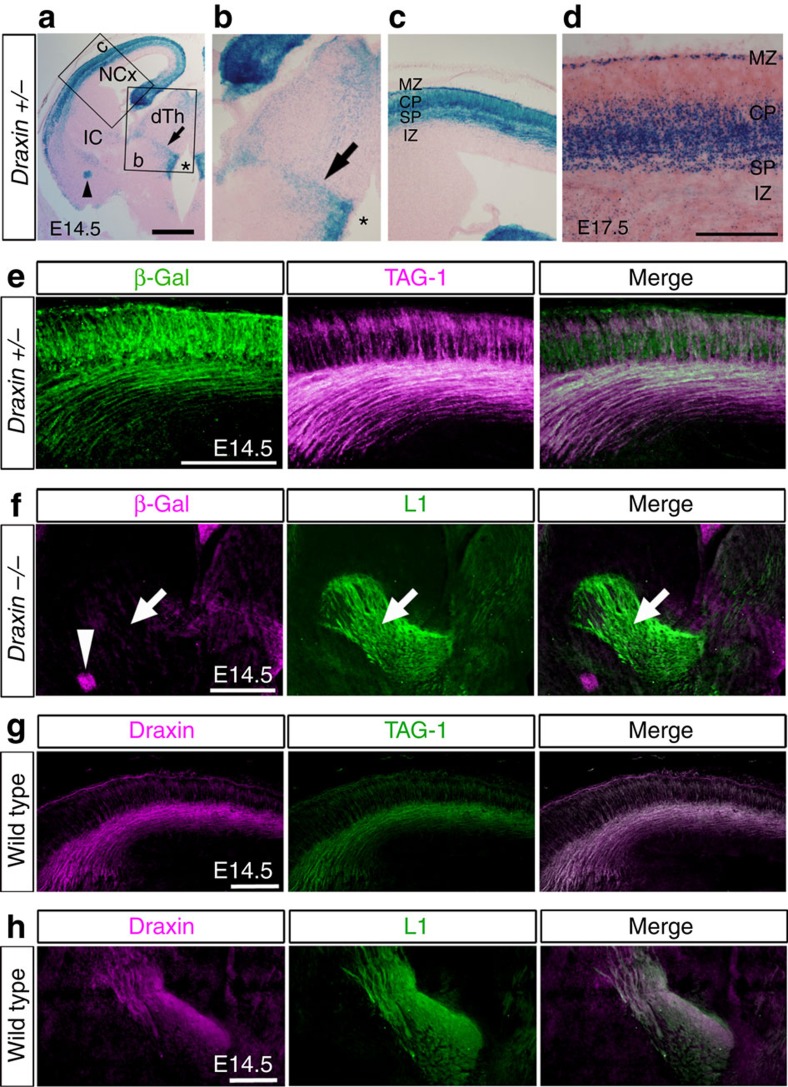
*Draxin* expression during corticofugal and thalamocortical development. (**a**–**c**) *draxin* expression visualized with β-gal staining in coronal sections of E14.5 *draxin*^*+/−*^ mice. *draxin* was strongly expressed in the neocortex and weakly expressed in the ventral telencephalon and the thalamus. Arrow, asterisk and arrowhead indicate *draxin* expression in the zona limitans intrathalamica, the ventricular zones of the ventral thalamus and a small portion of the amygdala, respectively. Scale bar, 500 μm. (**d**) *draxin* expression revealed with β-gal staining in the neocortex at E17.5. *draxin* expression was observed in early-born neurons, including in the subplate neurons. Scale bar, 200 μm. (**e**) Double immunostaining with β-gal and TAG-1 antibodies in *draxin*^*+/−*^ mice revealed that neocortical neurons express *draxin.* Scale bar, 200 μm. (**f**) Double immunostaining with β-gal and L1 antibodies in *draxin*^−/−^ mice. Even in *draxin*^−/−^ mice that show strong β-gal expression compared with that in *draxin*^*+/−*^ mice, β-gal expression was not visible in thalamocortical axons. Scale bar, 400 μm. (**g**,**h**) Double immunostaining with draxin/TAG-1 and draxin/L1 antibodies showed the presence of draxin proteins in the corticofugal and thalamocortical axons. Scale bars, 200 μm.

To investigate the expression pattern of *draxin* during the development of corticofugal and thalamocortical axons, we performed β-galactosidase (β-gal) staining on the brains of *draxin*^*+/−*^ mice, in which the second exon containing the ATG start codon was replaced with the *β-gal* gene^12^. At E14.5, *draxin* was strongly expressed in the neocortex (Fig. 3a,c) and was weakly expressed in the ventral telencephalon and thalamus (Fig. 3a,b). In the ventral telencephalon, *draxin* expression was observed in the corridor cells (Supplementary Fig. 3a). *draxin* expression was also observed in the zona limitans intrathalamica (the border between the dorsal and ventral thalamus; Fig. 3a,b, arrows), the ventricular zones of the ventral thalamus (Fig. 3a,b, asterisks) and the amygdala (Fig. 3a, arrowhead). Furthermore, β-gal staining at E17.5 clearly showed *draxin* expression in the early-born neurons, deep cortical plate, subplate and marginal zone of the neocortex (Fig. 3d). Double immunostaining against β-gal and TAG-1 or L1 at E14.5 revealed that *draxin* is strongly expressed in the corticofugal neurons, but not in the thalamocortical neurons (Fig. 3e,f). Consistent with this result, we confirmed with *in situ* hybridization that *draxin* messenger RNA is strongly expressed in the neocortex but not in the dorsal thalamus (Supplementary Fig. 3b). We next examined the distribution of draxin proteins in wild-type mice at E14.5 using a draxin antibody (Supplementary Fig. 3c). Double immunostaining against draxin and TAG-1 or L1 at E14.5 revealed the presence of draxin proteins in corticofugal and thalamocortical axons (Fig. 3g,h). These results suggest that draxin proteins on thalamocortical axons are mainly provided by diffusion from other regions including the corticofugal neurons.

**Figure 4:**
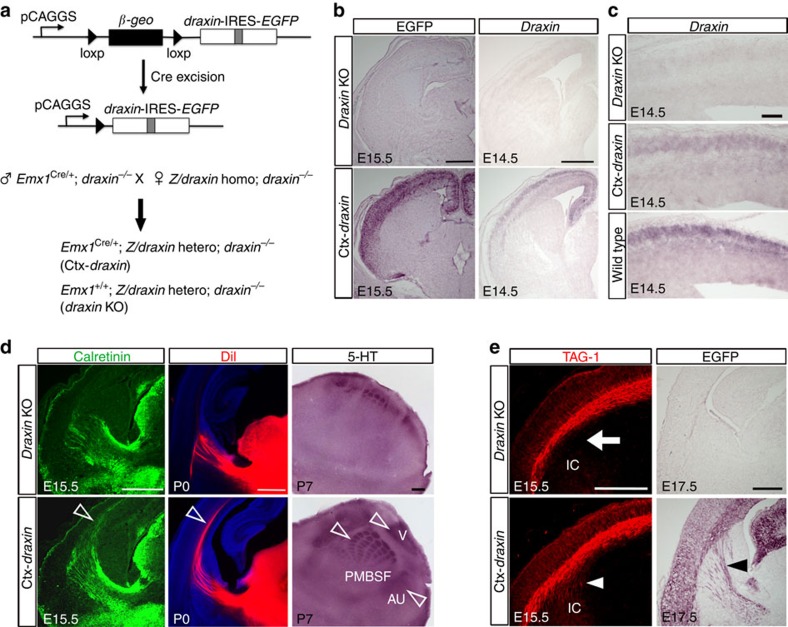
*Draxin* expression in the neocortex is critical for corticofugal and thalamocortical development. (**a**) Schematic of the Z/*draxin* transgene. Specific expression of *draxin* in the neocortex of *draxin*^−/−^ mice (*Emx1*^Cre/+^;Z/*draxin hetero*;*draxin*^−/−^) is achieved by crossing Z/*draxin homo*;*draxin*^−/−^ mice with *Emx1*^Cre/+^;*draxin*^−/−^ mice. In this study, *Emx1*^Cre/+^;Z/*draxin hetero*;*draxin*^−/−^ mice are referred to as Ctx-*draxin* mice and *Emx1*^+/+^;Z/*draxin hetero*;*draxin*^−/−^ mice are referred to as *draxin* knockout (KO) mice. (**b**) Immunostaining against EGFP and *in situ* hybridization for *draxin* in coronal sections of Ctx-*draxin* mice showed the specific expression of EGFP and *draxin* mRNA in the neocortex. Scale bars, 500 μm. (**c**) *draxin* mRNA expression detected with *in situ* hybridization in the neocortex of *draxin* KO, Ctx-*draxin* and wild-type mice. Scale bar, 100 μm. (**d**) Coronal or tangential sections of *draxin* KO and Ctx-*draxin* mice stained with calretinin or 5-HT antibodies. Coronal sections of *draxin* KO and Ctx-*draxin* mice with DiI injected into the dorsal thalamus. The thalamocortical phenotype was rescued in Ctx-*draxin* mice (*n*=5 for each condition). Open arrowheads indicate thalamocortical axons in the neocortex. Scale bars, 500 μm. (**e**) Coronal sections of *draxin* KO and Ctx-*draxin* mice stained with TAG-1 or EGFP antibodies. In *draxin* KO mice, TAG-1-positive axons did not extend into the internal capsule (arrow). This corticofugal phenotype was rescued in Ctx-*draxin* mice (arrowhead, *n*=4 for each condition). Scale bars, 500 μm.

Importantly, the thalamocortical phenotype of *draxin*^−/−^ mice is very similar to that of conditional mutant mice lacking corticofugal axons^10^. This fact suggests that *draxin* may be involved in establishing reciprocal interactions between corticofugal and thalamocortical axons. As *draxin* is strongly expressed in early-born neurons of the neocortex, draxin secreted from the neocortical neurons may be essential for thalamocortical axon development. To test this hypothesis, we performed rescue experiments using the Z/*draxin* transgenic mice, in which *draxin* and *EGFP* are co-expressed as a result of Cre-mediated recombination (Supplementary Fig. 4a–c)^26^. Induction of *draxin* expression in the neocortex of *draxin*^−/−^ (Ctx-*draxin*) mice was achieved by crossing Z/*draxin homo*;*draxin*^−/−^ mice with *Emx1*^Cre/+^;*draxin*^−/−^ mice, which show specific Cre expression in the dorsal telencephalon, but not in the ventral telencephalon and the thalamus (Fig. 4a)^27^,^28^. Immunostaining against enhanced green fluorescent protein (EGFP) and *in situ* hybridization for *draxin* showed the specific expression of EGFP and *draxin* transcripts in the dorsal telencephalon of Ctx-*draxin* mice (Fig. 4b). Furthermore, we investigated the expression levels of *draxin* in the neocortex of Ctx-*draxin* and wild-type mice at E14.5. We found that *draxin* expression in the neocortex of Ctx-*draxin* mice was lower than that in wild-type mice (Fig. 4c). Consistent with this result, we observed weak draxin immunoreactivity in the neocortex of Ctx-*draxin* mice compared with that of wild-type mice (Supplementary Fig. 4d,e). These results indicate that *draxin* expression was specifically induced in the neocortex of Ctx-*draxin* mice, and that Ctx-*draxin* mice have physiological expression levels of *draxin* mRNA and its proteins. To examine the trajectories of thalamocortical axons in Ctx-*draxin* mice, we performed immunostaining against calretinin and injected DiI into the neocortex. We found that thalamocortical axons extended into the neocortex of Ctx-*draxin* mice (Fig. 4d, open arrowheads). In addition, immunostaining with a 5-HT antibody also revealed that thalamocortical projections targeting the neocortex were sufficiently rescued in Ctx-*draxin* mice (Fig. 4d, open arrowheads). Next, using immunostaining against TAG-1 at E15.5, we examined whether the corticofugal phenotype is rescued in Ctx-*draxin* mice. In *draxin* knockout mice, TAG-1-positive axons did not extend into the internal capsule (Fig. 4e, arrow). In contrast, we found that TAG-1-positive axons in Ctx-*draxin* mice extended into the internal capsule (Fig. 4e, arrowhead). In addition, we observed corticofugal axons entering the internal capsule of Ctx-*draxin* mice using immunostaining against EGFP at E17.5 (Fig. 4e, arrowhead). These results demonstrate that corticofugal projections into the internal capsule were sufficiently rescued in Ctx-*draxin* mice. Thus, these data suggest that draxin from the neocortical neurons is sufficient for the normal development of corticofugal and thalamocortical axons.

### Draxin has an outgrowth-promoting effect on thalamic axons

**Figure 5:**
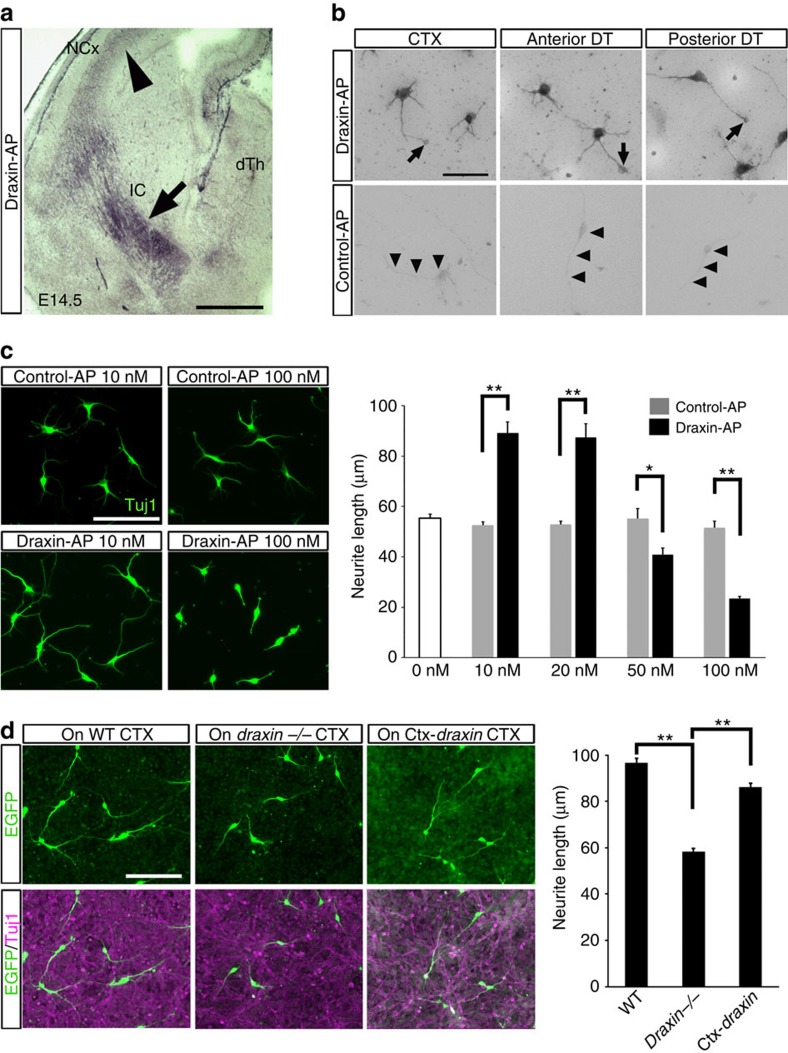
Outgrowth-promoting effects of draxin on thalamic axons. (**a**) Draxin-AP binding in coronal sections from E14.5 brains of wild-type mice. The draxin-AP signal was observed in the intermediate zone of the neocortex (arrowhead) and the internal capsule (arrow). Scale bar, 500 μm. (**b**) Draxin-AP bound to the majority of neurites extending from dissociated neurons of the neocortex (CTX), anterior dorsal thalamus (anterior DT) and posterior dorsal thalamus (posterior DT) of E14.5 wild-type mice. Arrows indicate draxin-AP binding to the growth cones of dissociated neurons. In contrast, control-AP did not bind to neurites from these neurons (arrowheads). Scale bar, 50 μm. (**c**) Dissociated cultures of thalamic neurons with different concentrations of draxin-AP proteins showed that neurite outgrowth was promoted by low draxin concentrations and inhibited by high draxin concentrations. Error bars are s.e.m. (*n*=5 independent experiments). *P<0.05 and **P<0.01 by Welch's *t*-test. Scale bar, 100 μm. (**d**) Dissociated cultures of thalamic neurons on neocortical neurons prepared from wild-type, *draxin*^−/−^ and Ctx-*draxin* mice. Neurite outgrowth was reduced in thalamic neurons cultured on *draxin*^−/−^ neocortical neurons compared with that in thalamic neurons cultured on wild-type neocortical neurons. This decreased neurite outgrowth was significantly rescued when thalamic neurons were cultured on Ctx-*draxin* neocortical neurons. Error bars are s.e.m. (*n*=3 independent experiments). **P<0.01 by one-way analysis of variance followed by Tukey's honest significance test. Scale bars, 100 μm.

**Figure 6:**
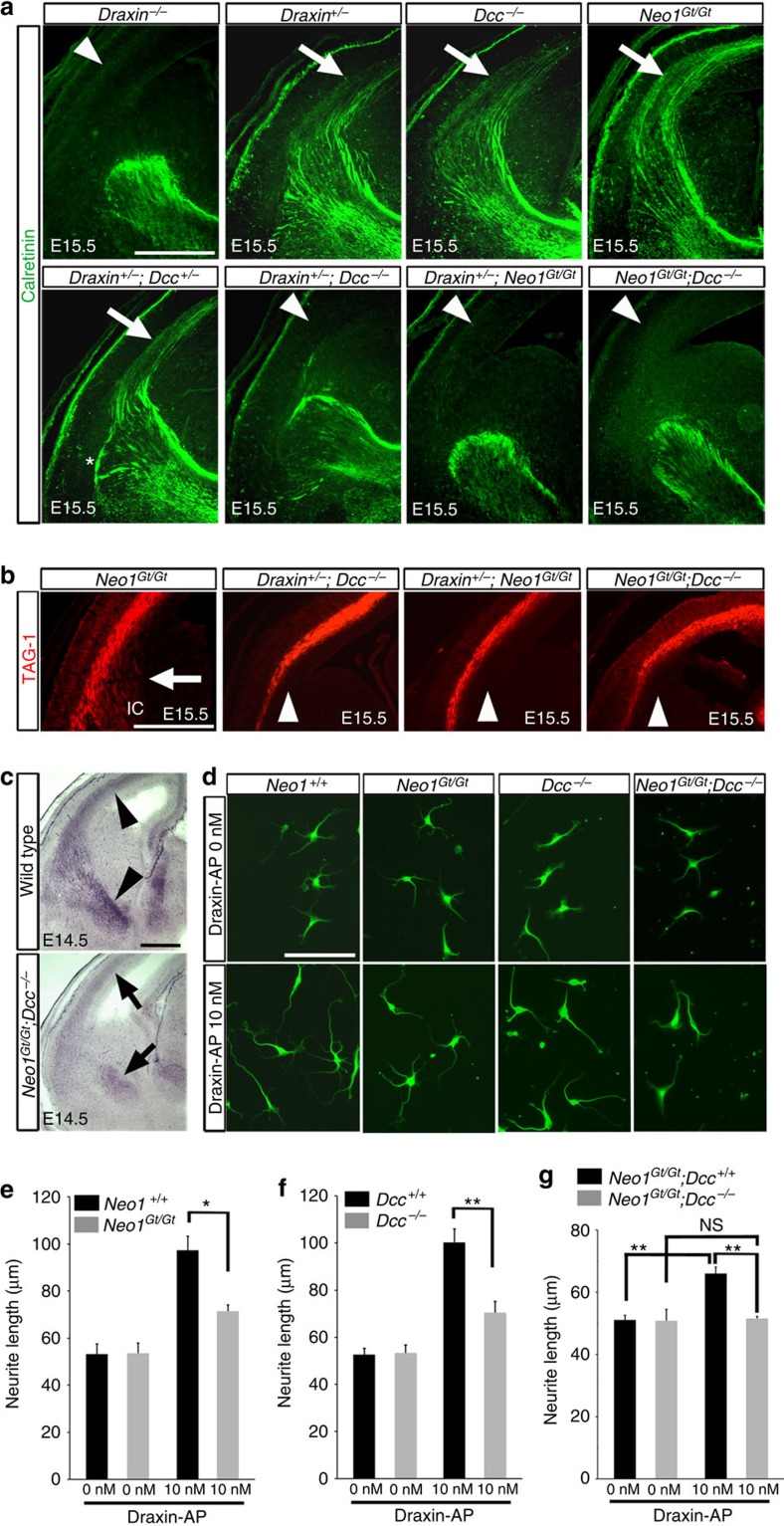
DCC and Neo1 are required for draxin receptor signalling. (**a**) Coronal sections from E15.5 brains of *draxin*^−/−^, *draxin*^*+/−*^, *Dcc*^−/−^, *Neo1*^Gt/Gt^, *draxin*^*+/−*^;*Dcc*^*+/−*^, *draxin*^*+/−*^;*Dcc*^−/−^, *draxin*^*+/−*^;*Neo1*^Gt/Gt^ and *Neo1*^Gt/Gt^;*Dcc*^−/−^ mice stained with a calretinin antibody. Thalamocortical projections into the neocortex were disrupted in *draxin*^*+/−*^;*Dcc*^−/−^, *draxin*^*+/−*^;*Neo1*^Gt/Gt^ and *Neo1*^Gt/Gt^;*Dcc*^−/−^ mice (arrowheads), but were not affected in *draxin*^*+/−*^, *Dcc*^−/−^ and *Neo1*^Gt/Gt^ mice (arrows). An asterisk indicates the misrouted thalamocortical axons. Scale bar, 500 μm. (**b**) Coronal sections from E15.5 brains of *Neo1*^Gt/Gt^, *draxin*^*+/−*^;*Dcc*^−/−^, *draxin*^*+/−*^;*Neo1*^Gt/Gt^ and *Neo1*^Gt/Gt^;*Dcc*^−/−^ mice stained with a TAG-1 antibody. TAG-1-positive axons of the *Neo1*^Gt/Gt^ mice extended into the internal capsule (arrow). In contrast, TAG-1-positive axons of *draxin*^*+/−*^;*Dcc*^−/−^, *draxin*^*+/−*^;*Neo1*^Gt/Gt^ and *Neo1*^Gt/Gt^;*Dcc*^−/−^ mice did not extend into the internal capsule (arrowheads, *n*=4 for each genotype). (**c**) Draxin-AP binding in coronal sections from E14.5 brains of wild-type and *Neo1*^Gt/Gt^;*Dcc*^−/−^ mice. Draxin-AP binding in the intermediate zone of the neocortex and the internal capsule in *Neo1*^Gt/Gt^;*Dcc*^−/−^ mice (arrows) was weaker than that in wild-type mice (arrowheads, *n*=3 for each genotype). Scale bar, 500 μm. (**d**–**g**) Dissociated cultures of thalamic neurons from *Dcc*^−/−^, *Neo1*^Gt/Gt^ and *Neo1*^Gt/Gt^;*Dcc*^−/−^ mice showed that DCC and Neo1 are necessary for the outgrowth-promoting effects of draxin. Scale bar, 100 μm. Quantification of the neurite outgrowth in *Neo1*^Gt/Gt^ (**e**) and *Dcc*^−/−^ (**f**) neurons in the presence of draxin-AP proteins. Error bars are s.e.m. (*n*=4 independent experiments). *P<0.05 and **P<0.01 by Welch's *t*-test. (**g**) Quantification of the neurite outgrowth in *Neo1*^Gt/Gt^;*Dcc*^−/−^ neurons in the presence of draxin-AP proteins. Error bars are s.e.m. (*n*=4 independent experiments). **P<0.01 and NS (not significant) by one-way analysis of variance followed by Tukey's honest significance test.

We hypothesized that draxin secreted from neocortical neurons controls the guidance of thalamocortical axons through the draxin receptors expressed in thalamic neurons. To test this hypothesis, we examined the distribution of draxin receptors in the brain at E14.5 with a binding assay based on the alkaline phosphatase (AP)-tagged draxin protein (draxin-AP)^12^. Draxin-AP binding was observed in the intermediate zone of the neocortex and in the internal capsule (Figs 5a and 6c). In contrast, control-AP binding was not observed in the brain sections (Supplementary Fig. 5a). These results suggest that draxin binds to corticofugal and thalamocortical axons, because at E14.5 the intermediate zone of the neocortex and the internal capsule mainly contain neocortical and thalamocortical axons, respectively. Next, we performed a draxin-AP-binding assay on dissociated neurons of the neocortex and the dorsal thalamus, to assess potential differences in the draxin-binding affinity of these neurons. The dorsal thalamus was subdivided into anterior and posterior regions. We found that draxin-AP bound to the majority of neurites extending from the dissociated neurons of the neocortex, anterior dorsal thalamus and posterior dorsal thalamus (Fig. 5b). There were no significant differences among the growth cones of these neurons regarding draxin-AP binding (Supplementary Fig. 5b). These results suggest that draxin binds to neocortical and thalamic axons with a similar affinity.

To examine the effect of draxin on the outgrowth of thalamic axons, we cultured dissociated thalamic neurons with different concentrations of draxin-AP proteins. Thalamic neurons were cultured on coverslips coated with poly-L-lysine (PLL) and L1, because L1 promotes significantly higher neurite outgrowth than PLL or laminin/PLL. This enabled us to evaluate the effects of draxin more easily (Supplementary Fig. 5c). Interestingly, draxin inhibited neurite outgrowth at high concentrations but promoted neurite outgrowth at lower concentrations (Fig. 5c). To further investigate the potential contribution of draxin to thalamocortical axonal outgrowth, dissociated thalamic neurons were cultured on HEK293T cells stably expressing draxin (draxin-293 cells). We observed 86% increase in neurite outgrowth of thalamic neurons cultured on draxin-293 cells compared with that on control-293 cells (Supplementary Fig. 5d). These data raise the possibility that draxin has an outgrowth-promoting effect on thalamic axons at physiological concentrations. To test this possibility, we cultured thalamic neurons obtained from CAG-EGFP mice^29^ on a neocortical feeder layer prepared from wild-type, *draxin*^−/−^ or Ctx-*draxin* mice. We confirmed that EGFP expression level in thalamic neurons from CAG-EGFP mice were significantly higher than that in neocortical neurons from Ctx-*draxin* mice (Fig. 5d, right panels). This enabled us to measure the neurite length of thalamic neurons cultured on neocortical neurons from Ctx-*draxin* mice using immunostaining against EGFP. We found that neurite outgrowth was enhanced on wild-type neocortical neurons that endogenously express draxin compared with that on *draxin*^−/−^ neocortical neurons (Fig. 5d). Furthermore, neocortical neurons from Ctx-*draxin* mice, compared with those from *draxin*^−/−^ mice, significantly promoted the neurite outgrowth of thalamic neurons, although the neurite outgrowth of thalamic neurons was not completely rescued by culturing them on the neocortical neurons of Ctx-*draxin* mice compared with culturing them on those of wild-type mice (Fig. 5d). These results suggest that draxin from neocortical neurons promotes the outgrowth of thalamocortical axons *in vivo*.

### DCC and Neo1 are draxin receptors in thalamic neurons

We previously reported that draxin binds to the netrin-1 receptors DCC, UNC5s, DSCAM and Neo1 (ref. 30). DCC has been proposed as a functional receptor for draxin in neurons from the neocortex^30^ and the dorsal horn of the spinal cord^31^. All of these receptors are expressed in the dorsal thalamus^32^,^33^,^34^,^35^. However, the thalamocortical phenotype observed in *draxin*^−/−^ mice has not been previously reported in mutants of these receptors. Thus, there may be redundancies in the receptors that mediate draxin signalling in thalamocortical neurons. To assess the ligand–receptor relationship between draxin and these receptors in thalamocortical projections *in vivo*, we investigated genetic interactions by generating compound mutant mice (Table 1). The mutations of *Dcc*^36^, *Unc5a*^37^, *Unc5b*^38^ and *Dscam*^39^ are very strongly hypomorphic or null alleles, whereas the mutation of *Neo1* is a hypomorphic allele^40^,^41^. Previous studies have demonstrated that Neo1 protein levels are reduced by ∼90% in *Neo1*^Gt/Gt^ mice^42^. We confirmed that the amount of Neo1 protein was dramatically reduced in the thalamus of *Neo1*^Gt/Gt^ embryos at E14.5 (Supplementary Fig. 6a). Thalamocortical projections in the internal capsule of various mutants were analysed by immunostaining against calretinin (Fig. 6a) and L1 (Supplementary Fig. 6b) at E15.5. *draxin*^−/−^ mice had a severe phenotype, as most thalamocortical axons did not enter the neocortex, whereas the thalamocortical projections of *draxin*^*+/−*^, *Dcc*^−/−^ or *Neo1*^Gt/Gt^ mice did not reveal any significant defects. Mild pathfinding defects of thalamocortical axons were observed in 36% of *draxin*^*+/−*^;*Dcc*^*+/−*^ mice and 33% of *draxin*^*+/−*^;*Neo1*^Gt/+^ mice. In addition, all *draxin*^*+/−*^;*Dcc*^−/−^ mice and 75% of *draxin*^*+/−*^;*Neo1*^Gt/Gt^ mice mimicked the thalamocortical phenotype of *draxin*^−/−^ mice. In contrast, no significant defects were observed in the thalamocortical projections of *draxin/Unc5a*, *draxin/Unc5b* or *draxin/Dscam* compound mutant mice. These results suggest that DCC and Neo1 might play critical roles in the draxin-mediated signalling pathway *in vivo*. Importantly, double mutants for *Dcc* and *Neo1* displayed a severe phenotype, which resembled that of *draxin*^−/−^ mice, indicating the compensatory functions of DCC and Neo1 in draxin signalling. Furthermore, we performed immunostaining against TAG-1, to examine the phenotypic similarities in corticofugal projections between *draxin*^−/−^ and compound mutant mice. We observed no significant defects in TAG-1-positive corticofugal axons of *draxin*^*+/−*^, *Dcc*^−/−^ or *Neo1*^Gt/Gt^ mice at E15.5 (Fig. 6b). In contrast, TAG-1-positive axons of *draxin*^*+/−*^;*Dcc*^−/−^, *draxin*^*+/−*^;*Neo1*^Gt/Gt^ or *Neo1*^Gt/Gt^;*Dcc*^−/−^ mice did not extend into the internal capsule (Fig. 6b), which was very similar to the phenotype observed in *draxin*^−/−^ mice. Thus, our data revealed a high degree of similarity in thalamocortical and corticofugal phenotypes between *draxin*^−/−^ and *Neo1*^Gt/Gt^;*Dcc*^−/−^ mice.

We determined the binding affinity of draxin to Neo1 in transfected cells by generating a binding curve with a dissociation constant (*K*_d_) of 620 pM (Supplementary Fig. 6c), which is comparable with the *K*_d_ of the draxin–DCC interaction^30^. To investigate whether DCC and Neo1 are the primary receptors of draxin *in vivo*, we performed a draxin-AP-binding assay on brain sections obtained from *Neo1*^Gt/Gt^;*Dcc*^−/−^ mice at E14.5. We found that the draxin-AP signals was robustly reduced in the intermediate zone of the neocortex and the internal capsule in *Neo1*^Gt/Gt^;*Dcc*^−/−^ mice (Fig. 6c, arrows) compared with that in wild-type mice (Fig. 6c, arrowheads). The amounts of neocortical and thalamocortical axons in these regions were not greatly affected in *Neo1*^Gt/Gt^;*Dcc*^−/−^ mice at this stage (Fig. 6a,b and Supplementary Fig. 6b). These results indicate that draxin binding was reduced in the neocortical and thalamocortical axons of *Neo1*^Gt/Gt^;*Dcc*^−/−^ mice, suggesting that DCC and Neo1 are the primary binding partners for draxin *in vivo*.

To explore whether DCC and Neo1 on thalamic axons are required for the outgrowth-promoting effects of draxin, which seems to be critical for thalamocortical development, we cultured dissociated thalamic neurons from *Dcc*^−/−^, *Neo1*^Gt/Gt^ and *Neo1*^Gt/Gt^;*Dcc*^−/−^ mice (Fig. 6d-g). There was no significant difference in the neurite outgrowth from thalamic neurons of wild-type (*Dcc*^+/+^ and *Neo1*^+/+^), *Dcc*^−/−^, *Neo1*^Gt/Gt^ and *Neo1*^Gt/Gt^;*Dcc*^−/−^ embryos in the absence of draxin-AP proteins. However, in the presence of draxin-AP proteins, we observed 27% and 30% reductions in the neurite outgrowth from thalamic neurons in *Neo1*^Gt/Gt^ and *Dcc*^−/−^ embryos, respectively, compared with that in littermate controls (Fig. 6e,f). Moreover, the growth-promoting effect of draxin-AP was absent in thalamic neurons from *Neo1*^Gt/Gt^;*Dcc*^−/−^ embryos (Fig. 6g). These results, taken together with our *in vivo* data, suggest that DCC and Neo1 are essential components of draxin-mediated pathway that controls the development of thalamocortical projections. In contrast to the outgrowth-promoting effect of draxin-AP, the inhibitory effect on neurite outgrowth induced by high concentrations of draxin-AP was not completely abolished in the thalamic neurons from *Neo1*^Gt/Gt^;*Dcc*^−/−^ embryos (Supplementary Fig. 6d). This result suggests that additional receptor(s) may be necessary for the inhibitory effect of draxin.

### Draxin regulates neurite outgrowth of neocortical neurons

*In vitro* binding assay showed that draxin bound not only to thalamic axons but also to neocortical axons with a similar affinity (Fig. 5b and Supplementary Fig. 5b). To test the effect of draxin on the outgrowth of neocortical axons, we cultured dissociated neocortical neurons from wild-type mice at E14.5. Neocortical neurons were cultured on coverslips coated with L1/PLL, because L1 promoted neurite outgrowth significantly better than PLL or laminin/PLL (Supplementary Fig. 7a). We found that the neurite outgrowth in neocortical neurons was promoted by draxin at low concentrations and inhibited at higher concentrations (Supplementary Fig. 7b). It was previously reported that *Dcc* and *Neo1* are expressed in neocortical neurons^43^,^44^. To examine whether DCC and Neo1 are necessary for the draxin-mediated effects on neocortical neurons, we cultured dissociated neocortical neurons from *Neo1*^Gt/Gt^;*Dcc*^−/−^ mice in the presence of draxin-AP proteins. The growth-promoting effect of 10 nM draxin-AP was completely abolished in neocortical neurons from *Neo1*^Gt/Gt^;*Dcc*^−/−^ embryos, whereas the inhibitory effect of 100 nM draxin-AP was partially abolished (Supplementary Fig. 7c,d). Thus, draxin regulates the neurite outgrowth of both thalamic and neocortical neurons in a concentration-dependent manner. Furthermore, our data suggest that DCC and Neo1 are sufficient to mediate the growth-promoting effect of draxin in these neurons, whereas additional receptor(s) may be necessary for the inhibitory effect of draxin.

## Discussion

The handshake hypothesis, proposed by Molnar and colleagues^45^, postulates that corticofugal and thalamocortical axons meet in the internal capsule and follow each other from the internal capsule to the thalamus and neocortex, respectively. In fact, the experimental data in mutant mice lacking different types of transcription factors such as Tbr1 (refs 5, 6), Fez-like^8^,^9^, Pax6 (refs 5, 46, 47), Gbx2 (refs 5, 48) and Emx2 (refs 49, 50) suggest that corticofugal and thalamocortical axons depend on each other for their guidance. Furthermore, it was shown in conditional mutant mice lacking corticofugal axons that descending corticofugal axons are essential for guiding thalamocortical axons across the PSPB^10^. In a complementary study using conditional mutant mice lacking thalamocortical axons, it has been shown that thalamocortical axons are necessary to guide corticothalamic axons into the corridor and towards the thalamus^25^. Recently, it has been reported that the transmembrane protein Linx is expressed on corticofugal axons and is necessary for thalamocortical development^11^. Although numerous studies support the handshake hypothesis, molecular mechanisms governing the interaction of these axons remained unclear. In this study, we showed that draxin expressed in the neocortical neurons is critical for the normal development of corticofugal and thalamocortical axons.

**Figure 7:**
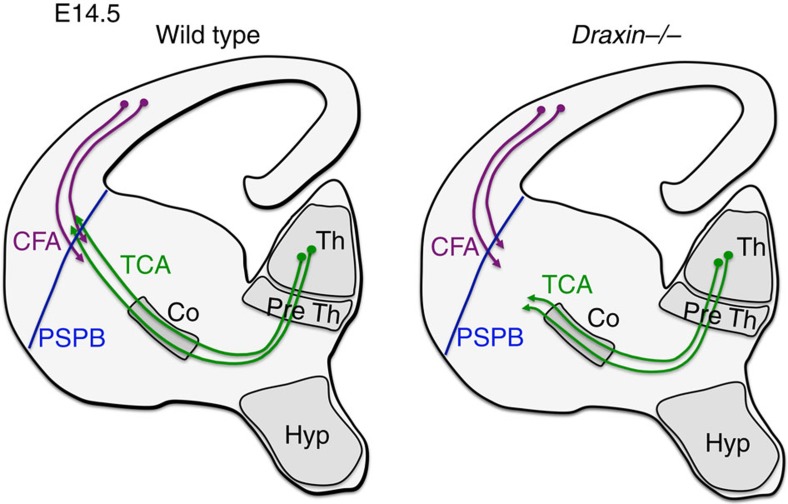
Summary of the *draxin*^−/−^ phenotype at E14.5. In *draxin*^−/−^ mice, thalamocortical axons did not cross the PSPB, whereas early corticofugal axons projected normally towards the internal capsule. CFA, corticofugal axons; Co, corridor cells; Hyp, hypothalamus; PreTh, prethalamus; PSPB, pallial-subpallial boundary. TCA, thalamocortical axons; Th, thalamus.

Several guidance molecules regulate the projection of thalamocortical axons through various decision points^1^. During embryonic development, thalamocortical axons first grow towards the hypothalamus through the prethalamus and then they turn laterally towards the internal capsule^51^ (Fig. 7). Slit1- and Slit2-mediated repulsion from the hypothalamus is required for this turning behaviour of thalamocortical axons^23^,^52^,^53^. In contrast to the hypothalamus, corridor cells in the ventral telencephalon express the axonal growth-promoting factor neuregulin-1 and thus establish a permissive environment for thalamocortical axons^18^. In the mutant for *NRG-1* or its receptor *ErbB4*, the majority of thalamocortical axons fail to progress normally through the corridor cells in the medial ganglionic eminence^18^. In contrast, in *draxin*^−/−^ mice thalamocortical axons elongated into the internal capsule normally and the formation of corridor cells was not impaired (Figs 2a,c and 7), although *draxin* was expressed in the corridor cells (Supplementary Fig. 3a). These facts suggest that *draxin* expression in the corridor cells may not be essential for thalamocortical development. This is also supported by the observation that draxin from neocortical neurons is sufficient for the normal development of thalamocortical axons (Fig. 4d). However, further investigations are needed using region-specific *draxin* conditional knockout mice, to clarify the precise role of *draxin* expression in the ventral telencephalon.

Importantly, thalamocortical axons in *draxin*^−/−^ mice did not cross the PSPB, whereas early corticofugal axons projected normally towards the internal capsule (Figs 2c and 7). This thalamocortical phenotype is very similar to that in conditional mutants lacking corticofugal axons^10^, suggesting the involvement of draxin in the interaction between corticofugal and thalamocortical axons. We demonstrated that the thalamocortical phenotype in *draxin*^−/−^ mice was rescued by the transgenic expression of *draxin* in the neocortex (Fig. 4), suggesting the importance of *draxin* expression in the neocortical neurons for this phenotype. In addition, our *in vitro* data showed that draxin promoted the axonal extension of thalamic neurons mediated by the DCC and Neo1 receptors (Figs 5c and 6d–g). Based on these results, we propose a model in which draxin secreted from the corticofugal axons promotes the growth of thalamocortical axons towards the neocortex and induces them to cross the PSPB. Thalamocortical axons stalled or grew slowly towards the external capsule in *draxin*^−/−^ mice (Figs 2c and 7), probably due to the loss of outgrowth-promoting effect of draxin generated by the corticofugal axons, although it is unclear why thalamocortical axons grow slowly towards the external capsule.

It should be noted that corticofugal and thalamocortical axons associated in an ectopic tract within the external capsule of *draxin*^−/−^ mice (Supplementary Fig. 1b). This close association of corticofugal and thalamocortical axons might be regulated by other short-range surface-bound molecules. Thus, we suggest that draxin functions as a long-range mediator of reciprocal interactions between corticofugal and thalamocortical axons. However, this observation might support that draxin provides a permissive environment at the PSPB for the normal development of corticofugal and thalamocortical axons. Importantly, draxin-AP strongly bound to the thalamocortical axons in the brain sections at E14.5, whereas we observed no specific AP signal in the cell populations at the PSPB such as the corridor cells in the medial ganglionic eminence (Figs 5a and 6c). This observation suggests that draxin is more likely to have direct functions for the guidance of thalamocortical axons. However, we cannot rule out the possibility that draxin is indirectly involved in the establishment of a permissive corridor at the PSPB. The importance of axon–axon interactions, which do not depend on axon–target interactions, has been well documented during the establishment of anterior–posterior and dorsal–ventral axes within the olfactory map^54^. The neocortex contains distinct populations of projection neurons that send axons to various areas, such as the contralateral cortex, thalamus and the spinal cord^22^. Self-organization by axon–axon interactions may be a critical mechanism for establishing complex neuronal circuits in the neocortex.

We showed that some of the corticofugal axons did not enter the internal capsule in *draxin*^−/−^ mice (Fig. 1b and Supplementary Fig. 1b) and this corticofugal phenotype was rescued by the transgenic expression of *draxin* in the neocortex (Fig. 4). A previous study showed that corticofugal axons enter the internal capsule even in the absence of thalamocortical axons^25^. These facts suggest that draxin might autonomously regulate the entering of corticofugal axons into the internal capsule. Accordingly, we showed that draxin bound to the neocortical axons and regulated neurite outgrowth *in vitro* (Supplementary Figs 5b and 7). Thus, these results are consistent with the idea that draxin functions in an autocrine manner to regulate the normal progression of corticofugal axons. On the other hand, we cannot exclude the possibility that primary defects in thalamocortical projections cause secondary pathfinding errors in corticofugal axons, because the guidance of corticofugal and thalamocortical axons depend on each other. Thalamocortical axons misprojecting towards the external capsule could misguide corticofugal axons to the external capsule, resulting in the reduction of corticofugal axons that enter the internal capsule. Further studies are needed to clarify the role of *draxin* in corticofugal axon development.

It has been recently reported that Neo1 is a functional netrin-1 receptor, which acts in concert with DCC to direct commissural axons in the spinal cord^55^. Indeed, commissural axon guidance defects in *netrin-1*^−/−^ mutants are comparable to those in *Dcc* and *Neo1* double mutants^55^. In the forebrain, *netrin-1* is expressed in the ventral telencephalon and it is involved in the topographic establishment of thalamocortical projections^33^,^34^. However, *netrin-1* is not expressed in neocortical neurons and no obvious defect is observed during thalamocortical axon pathfinding in the internal capsule of *netrin-1*^−/−^ embryos. Importantly, *Neo1*^Gt/Gt^;*Dcc*^−/−^ mice showed a severe thalamocortical projection phenotype similar to that observed in *draxin*^−/−^ mice (Fig. 6). These facts suggest that draxin is a major ligand for Neo1- and DCC-mediated guidance of thalamocortical projections into the neocortex.

Our *in vitro* data suggest that low concentrations of draxin (10 nM) stimulate the neurite outgrowth of neocortical and thalamic neurons through the DCC and Neo1 receptors. We previously reported that 10 nM draxin inhibits neurite outgrowth from the neurons of the dorsal spinal cord^12^. How can draxin induce opposite growth behaviours in different neurons? A plausible mechanism, supported by previous studies on other axon guidance cues, is that different receptors or proteoglycans might be expressed on spinal cord neurons and neocortical/thalamic neurons. It has been shown that several axon guidance cues including netrin-1 and semaphorins (Sema) can act either as attractive or repulsive cues depending on the receptor complexes present on the growth cones^56^. For instance, netrin-1 binding to DCC induces attraction^57^, whereas this response is repulsive when both UNC5 and DCC are present^58^. In addition, the attractive and repulsive effects of Sema5A are controlled by different sulfated proteoglycans^59^. It should be noted that the effect of draxin on neurite outgrowth depends on its concentration (Fig. 5c and Supplementary Fig. 7b). We showed that DCC and Neo1 are sufficient to mediate the positive effects of draxin, but not its negative effects, suggesting that additional receptor(s) may be necessary for mediating the negative effects of draxin. Similar bimodal responses depending on the concentration have been observed in the case of ephrinA2 (ref. 60) and Shh^61^ on retinal ganglion cells, although extracellular and intracellular mechanisms underlying these bimodal effects remain unclear. In recent times, the crucial role of local protein synthesis has been shown in the concentration-dependent activities of axon guidance cues Sema3A and Sema3F^62^. To realize their repulsive guidance activities, low and high concentrations of Sema3A and Sema3F engage two distinct signal transduction pathways, a protein synthesis-dependent and -independent pathway. Importantly, DCC interacts with the translation machinery and functionally mediates translational regulation in response to netrin-1 (ref. 63). Thus, the concentration-dependent effects of draxin might require DCC-mediated local protein synthesis.

## Methods

### Mutant mice

Mice were treated according to protocols approved by the Committee on Animal Research at the University of Kumamoto. To obtain *draxin*^−/−^ brains, *draxin*^*+/−*^ males were crossed with *draxin*^*+/−*^ or *draxin*^−/−^ females, which were maintained in a mixed C57BL/6-CBA background^12^. We observed that the phenotypes in *draxin*^−/−^ mice were essentially identical in the F_1_ to F_6_ generations, which were backcrossed with C57BL/6N or ICR mice. *Dcc*^36^, *Neo1* (MMRRC:030400-MU)^40^,^41^, *Unc5a* (MMRRC:030749-MU)^37^, *Unc5b* (MMRRC:030410-MU)^38^ and *Dscam (RBRC:05290)*^39^ mutant mice and genotyping methods have been described previously. Male and female mice between 3 and 6 months old were used for breeding. All embryos and newborns examined in this study were collected regardless of sex.

### Generation of *draxin* transgenic mice

To generate the pZ/*draxin* expression construct, the floxed vector pCAG-loxP-βgeo-loxP, based on the pCALL construct^26^, was constructed using the pCAGGS vector^64^. Mouse *draxin* complementary DNA, together with an *IRES2-EGFP* (Clontech) sequence, were cloned downstream of the loxP-βgeo-loxP in this vector. Immunocytochemistry and western blot analysis using rabbit anti-draxin antibody (1:1,000)^12^ showed that transfection of HEK293T cells with the pZ/*draxin* construct induced Cre-dependent expression of draxin (Supplementary Fig. 4a,b). A corresponding uncropped western blot image was provided in Supplementary Fig. 8. The CAG-loxP-βgeo-loxP-*draxin*-*IRES2-EGFP* fragment was microinjected into the pronuclei of fertilized C57BL/6N oocytes to generate transgenic mice (CARD, Kumamoto University.). Routine genotyping was performed using PCR with the primers 5′-CAAAGGAATGCAAGGTCTGT-3′ and 5′-CATCCTTCAGCCCCTTGTTG-3′, which amplified 260 bp. We selected one transgenic line that exhibited significant β-geo expression levels in the brain. Emx1-Cre knock-in mice (RBRC01345) have been described previously^27^,^28^.

### Histological analyses

*In situ* hybridization and immunohistochemistry were performed as previously described^12^. For immunohistochemistry, we prepared 14- to 18-μm-thick cryostat and paraffin-embedded sections mounted onto glass slides and 50- to 100-μm-thick floating sections. The following primary antibodies were used: mouse anti-NF (2H3; Hybridoma Bank, 1:100), rabbit anti-5-HT (ImmunoStar, 1:50,000), rabbit anti-5-HT transporter (ImmunoStar, 1:10,000), rabbit anti-Tbr1 (Abcam, 1:200), mouse anti-chondroitin sulfate proteoglycan (Sigma, 1:500), mouse anti-MAP2 (Sigma, 1:1,000), rabbit anti-calretinin (Swant, 1:1,000), rabbit anti-calretinin (Millipore, 1:500), mouse anti-TAG-1 (4D7; Hybridoma Bank, 1:10), goat anti-TAG-1 (R&D Systems, 1:500), rabbit anti-β-gal (Cappel, 1:10,000), mouse anti-Islet1 (39.4D5; Hybridoma Bank, 1:200), rabbit anti-Nkx2-1 (TTF1; Biopot, 1:200), rat anti-GFP (Nacalai Tesque, 1:1,000), rat anti-L1 (Chemicon, 1:1,000), goat anti-netrin-G1 (R&D Systems, 1:100), rat anti-Ctip2 (Abcam, 1:1,000), rabbit anti-Foxp2 (Abcam, 1:10,000), goat anti-Brn2 (Santa Cruz Biotechnology, 1:300) and sheep anti-draxin (R&D Systems, 1:300). Primary antibodies were detected with Alexa-conjugated fluorescent secondary antibodies (Invitrogen, 1:500). For nonfluorescent detection, sections were incubated with biotinylated secondary antibodies and processed using the ABC histochemical method (Vector). β-Gal staining was performed on 16- to 20-μm-thick cryostat sections mounted onto glass slides. Sections were incubated with β-gal staining solution (1 mg ml^−1^ X-gal, 5 mM K_3_Fe(CN)_6_, 5 mM K_4_Fe(CN)_6_, 2 mM MgCl_2_ and PBS) for 10–16 h at 37 °C and were then counterstained with 1% eosin.

### Axon tracing

To label corticofugal axons, a 10% solution of DiI (Invitrogen) in *N*,*N*-dimethylformamide (Nacalai Tesque) was injected into the neocortex of the anaesthetized pups at postnatal day 0 (P0). The pups were perfused at P1 and the brains were postfixed by overnight immersion in 4% paraformaldehyde (PFA). To label thalamocortical axons, 4% PFA-fixed brains at P1 were bisected into hemispheres. Small crystals of DiI were placed into the medial face of the dorsal thalamus with an insect pin. For two-colour fluorescent tracing, DiI and 4-(4-(dihexadecylamino)styryl)-*N*-methylpyridinium iodide (Invitrogen) were injected in the neocortex and dorsal thalamus, respectively, of 4% PFA-fixed brains at E17.5. The brains were then incubated in PBS for 2**–**4 weeks at 37 °C. The labelled brains were embedded in 3% agarose and 100-μm-thick sections were cut on a Vibratome in the coronal plane. Sections were counterstained with Hoechst 33342 (Invitrogen).

### Draxin-AP binding assay

Control-AP (pAPtag-5 vector) and draxin-AP constructs were transfected into HEK293T cells (Riken BRC)^12^. After 4**–**5 days, the conditioned media was harvested and then concentrated with the Amicon Ultra-15 filter device (Millipore). In the conditioned media, draxin-AP was detected using western blotting with an anti-draxin antibody (1:1,000)^12^. Control- and draxin-AP concentrations in the conditioned medium were determined with the SensoLyte pNPP Secreted Alkaline Phosphatase Reporter Gene Assay Kit (AnaSpec). Draxin-AP binding to the dissociated neurons and brain sections was investigated as previously described^12^,^65^. We quantified draxin-AP signals on the growth cones of dissociated neurons from the neocortex, anterior dorsal thalamus and posterior dorsal thalamus. For the visualization of draxin-AP binding, neurons were stained with 5-bromo-4-chloro-3-indolyl phosphate and nitro blue tetrazolium for 12 or 24 h at room temperature. We calculated the normalized AP signal intensity, which was the signal intensity at the growth cones of dissociated neurons divided by the signal intensity of the background. In each condition, AP signals were measured for three neurons using the BZ-II analysis system (Keyence) and mean values were calculated. Statistical analyses were performed on the data obtained from three independent experiments.

### Dissociated culture from wild-type mice

Thalamic and neocortical neurons from E14.5 mouse embryos were dissociated using papain (Nacalai Tesque). We used the entire dorsal thalamus to prepare the dissociated culture of thalamic neurons, because anterior and posterior dorsal thalamic axons responded similarly to draxin. Dissociated neurons were plated at a density of 3 × 10^4^ cells per dish in four-well dishes on coverslips coated with PLL, PLL and laminin, or PLL and L1-Fc chimeric protein^66^. The cultures were maintained in Neurobasal medium, B27, glutamax-I and penicillin/streptomycin (Invitrogen) for 40 h with different concentrations of draxin-AP-conditioned medium. After culturing, neurons were stained with a neuron-specific beta-III tubulin (Tuj1) antibody (R&D Systems, 1:1,000). For the quantification of neurite length, photographs of randomly chosen fields were taken using an inverted fluorescence microscope (Keyence, Biorevo). Neurite length was measured as the distance between the tip of the longest neurite and the periphery of the cell body. In each condition, we performed the measurement in 30 neurons that could be distinguished from neighbouring neurons. We observed that ∼20**–**30% of the neurons had very short or no neurites independently of the culture conditions. To exclude these neurons from the quantitative analysis, 21 of the 30 neurons (70%) with the longest neurites were analysed using the BZ-II analysis system. Statistical analyses were conducted with the number of independent experiments and the graphs summarizing the results show average values from independent experiments.

### Dissociated culture on draxin-293 cells and neocortical neurons

To create stable HEK293T cells expressing the *draxin* gene, we used the Flp-In System (Invitrogen), which allows expression of a gene of interest in mammalian cells at a specific genomic location by FLP recombinase-mediated integration. Draxin expression in draxin-293 cells was confirmed by immunohistochemistry and western blotting using the draxin antibody (1:1,000)^12^. Draxin-293 or control-293 cells were dissociated with trypsin/EDTA (Invitrogen) and plated at a density of 5 × 10^5^ cells per dish in four-well dishes. Twenty-four hours later, dissociated thalamic neurons from wild-type mice at E14.5 were plated at a density of 3 × 10^4^ cells per dish onto the draxin-293 or control-293 cells. Cultures were grown for 40 h and then neurons were stained with a Tuj1 antibody. To establish a neocortical feeder layer, dorsal cortices of *draxin*^−/−^, wild-type or Ctx-*draxin* mice at E14.5 were dissociated with papain and plated at a density of 3 × 10^6^ cells per dish in four-well dishes. Two hours later, 3 × 10^4^ cells of dissociated thalamic neurons from CAG-EGFP mice at E14.5 were plated onto the neocortical feeder layer^29^. After culture for 24 h, neurons were double-stained with Tuj1 and GFP antibodies. Neurite outgrowth was analysed as described above.

### Dissociated culture from receptor mutant mice

To test the ability of *Dcc-* and/or *Neo1*-deficient neurons to respond to draxin, thalamic and neocortical neurons were dissected from these mutant embryos at E14.5 after genotyping by PCR and cultured for 40 h with draxin-AP-conditioned medium. *Dcc*^−/−^ or *Neo1*^Gt/Gt^ embryos were generated by crossing heterozygous parents. Double mutant embryos were generated from intercrosses of *Dcc*^*+/−*^; *Neo1*^Gt/Gt^ mice. Neurite outgrowth was analysed as described above.

### Western blot analysis

The dorsal thalamus from *Neo1*^+/+^ and *Neo1*^Gt/Gt^ embryos at E14.5 was dissected and lysed using lysis buffer (50 mM HEPES pH 7.6, 150 mM NaCl, 0.1% Triton X-100) supplemented with protease inhibitors (1 mM phenylmethylsulfonyl fluoride (Sigma), 10 μg ml^−1^ aprotinin (Sigma) and 10 μg ml^−1^ leupeptin (Sigma)). The lysate was centrifuged 15,000 r.p.m. at 4 °C for 20 min and was separated by SDS–PAGE. Neo1 (Santa Cruz Biotechnology, 1:100) and glyceraldehydes 3-phosphate dehydrogenase (Sigma, 1:1,000) antibodies were used for western blot analysis. Uncropped western blot images for Supplementary Fig. 6a were provided in Supplementary Fig. 8.

### Dissociation constant for draxin–Neo1 binding

Saturation binding curves and Scatchard analyses of draxin-AP binding to Neo1 were performed and the *K*_d_ value was determined as previously described^67^. Briefly, *Neo1*-transfected HEK293T cells were incubated with different concentrations of draxin-AP-conditioned medium for 90 min at room temperature with occasional gentle stirring. Cells were then washed six times with HBAH buffer (150 mM NaCl, 20 mM HEPES pH 7.0, 0.5 mg ml^−1^ BSA, 0.1% NaN_3_) and lysed with lysis buffer (10 mM Tris-HCl pH 8.0, 1% Triton X-100). Cell lysates were collected and centrifuged to collect supernatant, which was heated at 65 °C for 15 min. An equal volume of 2 × AP buffer (6.7 mg ml^−1^ p-nitrophenyl phosphate, 1 mM MgCl_2_, 2 M diethanol amine pH 9.8) was then added in each tube. Following a 1-h incubation at 37 °C, absorbance was measured at 405 nm.

## Additional information

**How to cite this article:** Shinmyo, Y. *et al.* Draxin from neocortical neurons controls the guidance of thalamocortical projections into the neocortex. *Nat. Commun.* 6:10232 doi: 10.1038/ncomms10232 (2015).

## Supplementary Material

Supplementary Information Supplementary Figures 1-8.

## Figures and Tables

**Table 1:** Frequencies of thalamocortical phenotypes in the compound mutants.

**Genotype**	**Normal** ***n*** **(%)**	**Mild** ***n*** **(%)**	**Severe** ***n*** **(%)**
*draxin*^−/−^ (*n*=8)	0	0	8 (100%)
*draxin*^*+/−*^ (*n*=8)	8 (100%)	0	0
*Dcc*^−/−^ (*n*=8)	8 (100%)	0	0
*draxin*^*+/−*^;*Dcc*^*+/−*^ (*n*=11)	7 (64%)	4 (36%)	0
*draxin*^*+/−*^; *Dcc*^−/−^ (*n*=8)	0	0	8 (100%)
*Neo1*^*Gt/Gt*^ (*n*=8)	8 (100%)	0	0
*draxin*^*+/−*^;*Neo1*^*Gt/+*^ (*n*=9)	6 (67%)	3 (33%)	0
*draxin*^*+/−*^;*Neo1*^*Gt/Gt*^ (*n*=8)	0	2 (25%)	6 (75%)
*Unc5a*^−/−^ (*n*=8)	8 (100%)	0	0
*draxin*^*+/−*^;*Unc5a*^*+/−*^ (*n*=8)	8 (100%)	0	0
*draxin*^*+/−*^;*Unc5a*^−/−^ (*n*=8)	8 (100%)	0	0
*Unc5b*^−/−^ (*n*=0)	Embryonic lethal (E10)		
*draxin*^*+/−*^;*Unc5b*^*+/−*^ (*n*=8)	8 (100%)	0	0
*Dscam*^−/−^ (*n*=8)	8 (100%)	0	0
*draxin*^*+/−*^;*Dscam*^*+/−*^ (*n*=8)	8 (100%)	0	0
*draxin*^*+/−*^;*Dscam*^−/−^ (*n*=8)	8 (100%)	0	0
*Neo1*^*Gt/Gt*^*;Dcc*^−/−^ (*n*=6)	0	0	6 (100%)

*draxin*^*+/−*^;*Dcc*^−/−^, *draxin*^*+/−*^;*Neo1*^Gt/Gt^ and *Neo1*^Gt/Gt^;*Dcc*^−/−^ mice exhibited severe thalamocortical projection defects that resembled those in *draxin*^−/−^ mice.
