# Supplementary material for: Draxin from neocortical neurons controls the guidance of thalamocortical projections into the neocortex
Source: Nat Commun. 2015 Dec 14;6:10232. doi: 10.1038/ncomms10232 (PMC4682175; doi:10.1038/ncomms10232)
Supplement: Supplementary Information — Supplementary Figures 1-8. [file ncomms10232-s1.pdf]

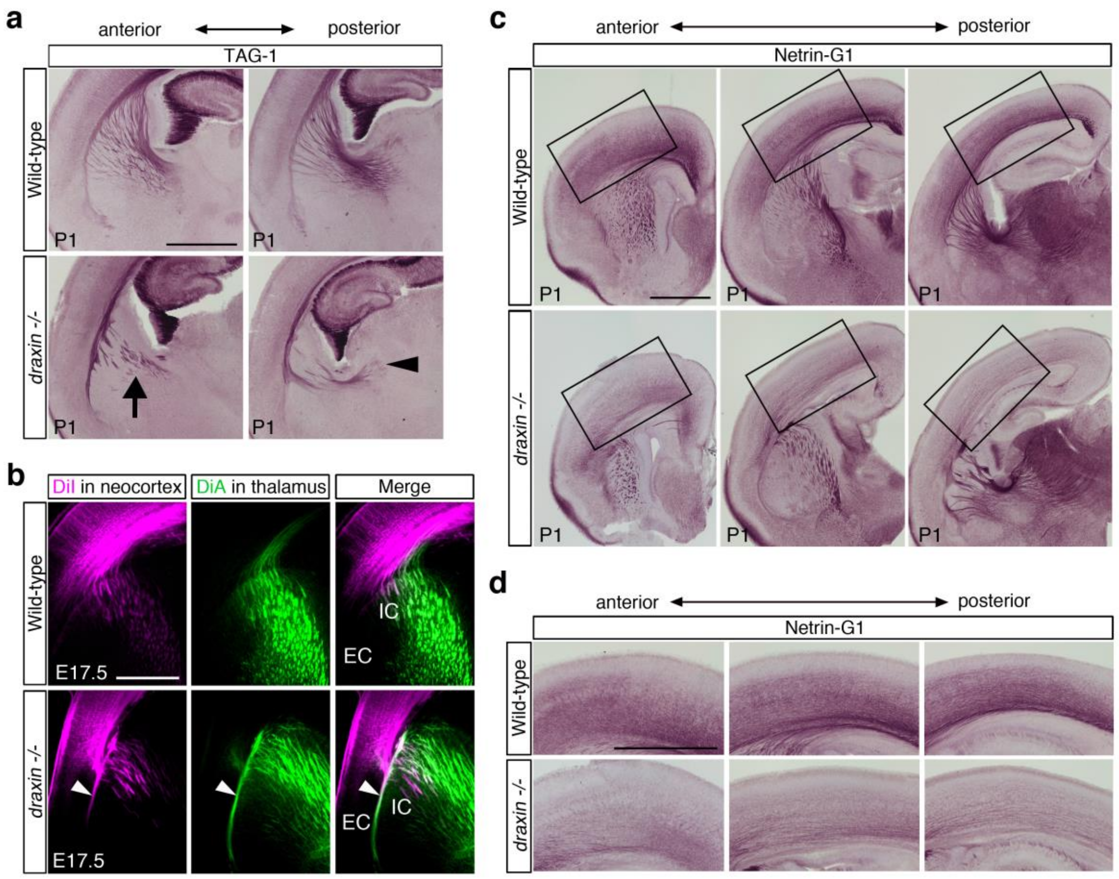

Supplementary Figure 1. Corticofugal and thalamocortical phenotypes in *draxin*$^{-/-}$ mice.
(**a**) Serial coronal sections of P1 brains from wild-type and *draxin*$^{-/-}$ mice stained with a TAG-1 antibody. The number of TAG-1-positive axons entering the internal capsule was reduced in *draxin*$^{-/-}$ mice (arrow). Some of the TAG-1-positive axons entered the internal capsule and reached the thalamus in *draxin*$^{-/-}$ mice (arrowhead, *n* = 4 for each genotype). Scale bar, 1 mm. (**b**) Coronal sections from E17.5 brains of wild-type and *draxin*$^{-/-}$ mice with a DiI injection in the neocortex and a DiA injection in the thalamus. Corticofugal and thalamocortical axons were apposed in the internal capsule and in an ectopic pathway in the external capsule (arrowheads) of *draxin*$^{-/-}$ mice (*n* = 10 for each genotype). Scale bar, 500 µm. (**c**,**d**) Serial coronal sections of P1 brains from wild-type and *draxin*$^{-/-}$ mice stained with a netrin-G1 antibody. The boxed areas in (**c**) are enlarged in (**d**). The number of thalamocortical axons was strongly reduced throughout the neocortex of *draxin*$^{-/-}$ mice (*n* = 4 for each genotype). Scale bars, 1 mm. IC, internal capsule; EC, external capsule.

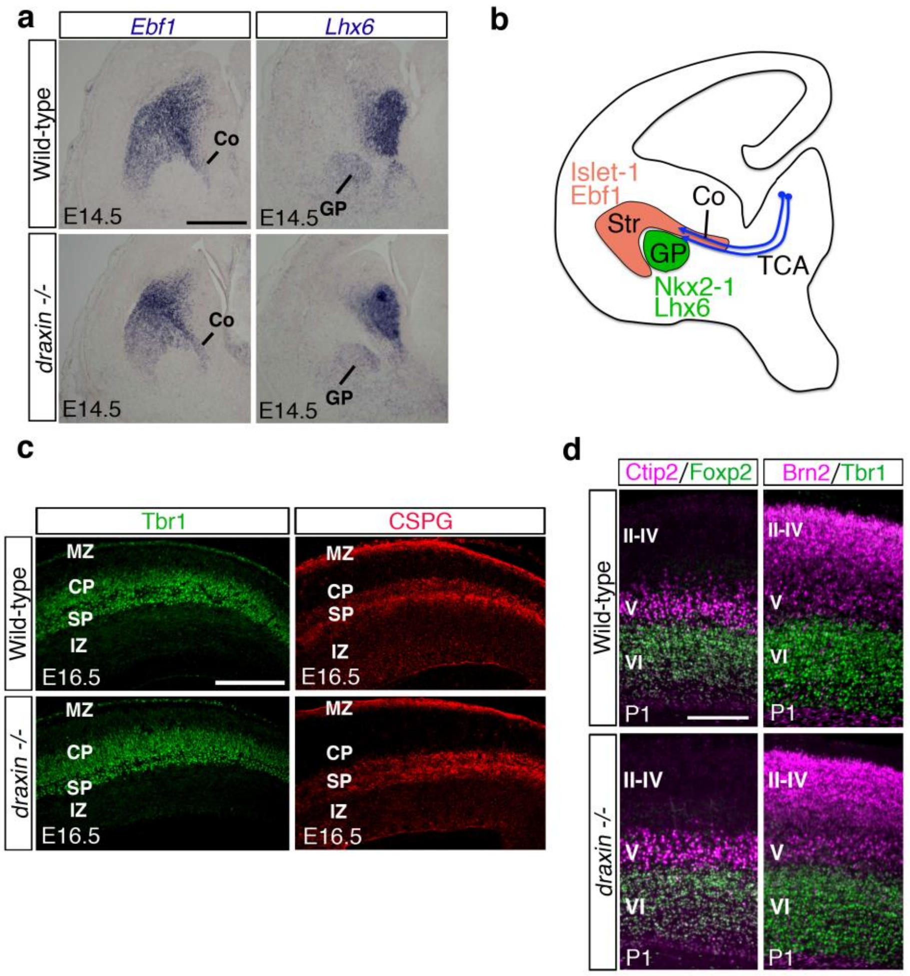

Supplementary Figure 2. Corridor cells and neocortical layers are formed normally in *draxin*$^{-/-}$ mice.

(**a**) *Ebf1* and *Lhx6* expression detected with *in situ* hybridization in coronal sections from the E14.5 brains of wild-type and *draxin*$^{-/-}$ mice. The corridor and the globus pallidus cells were formed normally in *draxin*$^{-/-}$ mice (n = 8 for each genotype). Scale bar, 500 μm. (**b**) Molecular markers in the ventral telencephalon. (**c**) Coronal sections from E16.5 brains of wild-type and *draxin*$^{-/-}$ mice stained with Tbr1 and CSPG antibodies. These markers were expressed normally in the neocortex of *draxin*$^{-/-}$ mice (n = 8 for each genotype). Scale bar, 300 μm. (**d**) Double immunostaining against Ctip2/Foxp2 and Brn2/Tbr1 in coronal sections from P1 brains of wild-type and *draxin*$^{-/-}$ mice. Expression patterns of these markers were not affected in *draxin*$^{-/-}$ mice (n = 4 for each genotype). Scale bar, 200 μm. Co, corridor cells; GP, globus pallidus; Str, striatum; TCA, thalamocortical axons; MZ, marginal zone; CP, cortical plate; SP, subplate; IZ, intermediate zone.

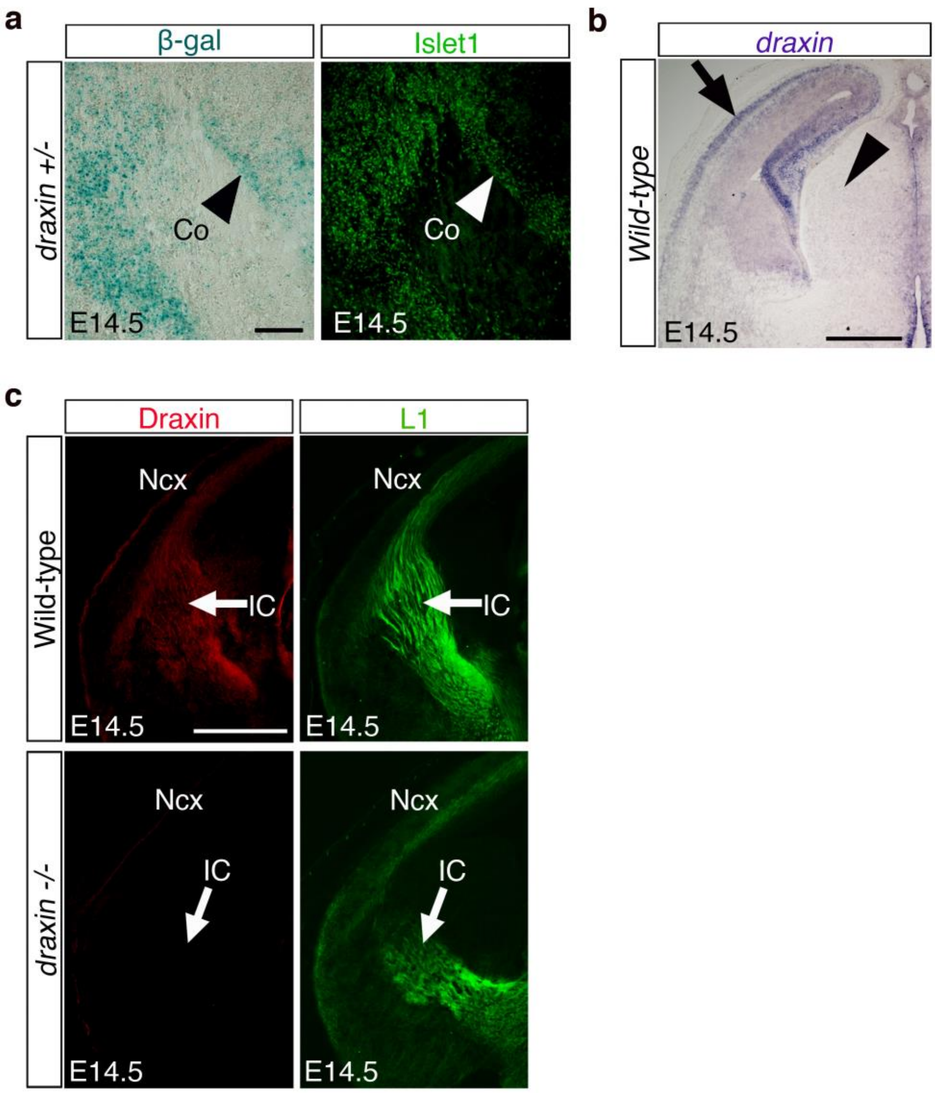

Supplementary Figure 3. *draxin* mRNA and protein expression patterns during corticofugal and thalamocortical development.

(**a**) β-gal staining and immunostaining against Islet1 in neighboring coronal sections of E14.5 *draxin*$^{+/-}$ mice. Note that *draxin* is expressed in the corridor cells (arrowheads). Scale bar, 100 µm. (**b**) *In situ* hybridization for *draxin* in the coronal sections of E14.5 wild-type mice. *draxin* is strongly expressed in the neocortex (arrow), but not in the dorsal thalamus (arrowhead). Scale bar, 500 µm. (**c**) Double immunostaining with draxin and L1 antibodies in wild-type and *draxin*$^{-/-}$ mice. Note that draxin proteins were observed in the corticofugal and thalamocortical axons of wild-type mice, but were not detectable in *draxin*$^{-/-}$ mice. Scale bar, 500 µm. Co, corridor cells; Ncx, neocortex; IC, internal capsule.

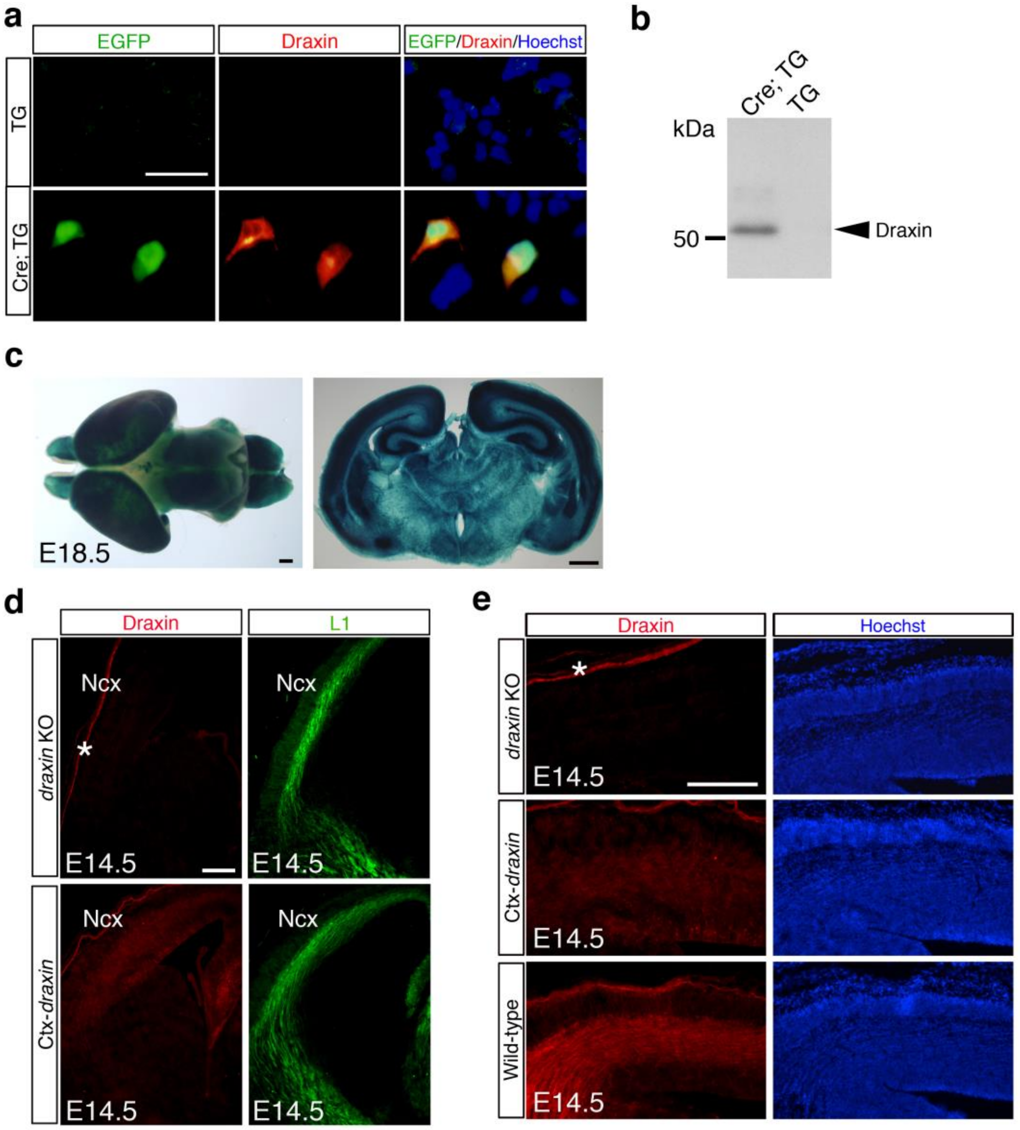

Supplementary Figure 4. Generation of *draxin* conditional transgenic mice.

(**a**) Cre-dependent expression of draxin in HEK293T cells. HEK293T cells transfected with pMCX-Cre and pZ/*draxin* (Cre; TG) or only pZ/*draxin* (TG) were stained with a draxin antibody and Hoechst 33342. EGFP and draxin were expressed in HEK293T cells transfected with Cre; TG, whereas no expression was observed in HEK293T cells transfected with only TG. Scale bar, 50 μm. (**b**) Western blot analysis using a draxin antibody revealed that draxin proteins were present in the supernatant of HEK293T cells transfected with Cre; TG. (**c**) β-geo expression in the E18.5 brains of Z/*draxin* transgenic mice. We selected a transgenic line that expressed β-geo at a significant level in the brain. Scale bars, 500 μm. (**d,e**) Coronal sections from E14.5 brains of *draxin* KO and Ctx-*draxin* mice stained with a draxin antibody, a L1 antibody, and Hoechst 33342. Draxin immunoreactivity was detected in the neocortex of Ctx-*draxin* mice, but not in that of *draxin* KO mice (**d**). We observed weak draxin immunoreactivity in the neocortex of Ctx-*draxin* mice compared with that of wild-type mice (**e**). Asterisks indicate nonspecific staining in the meninges. Scale bars, 200 μm.

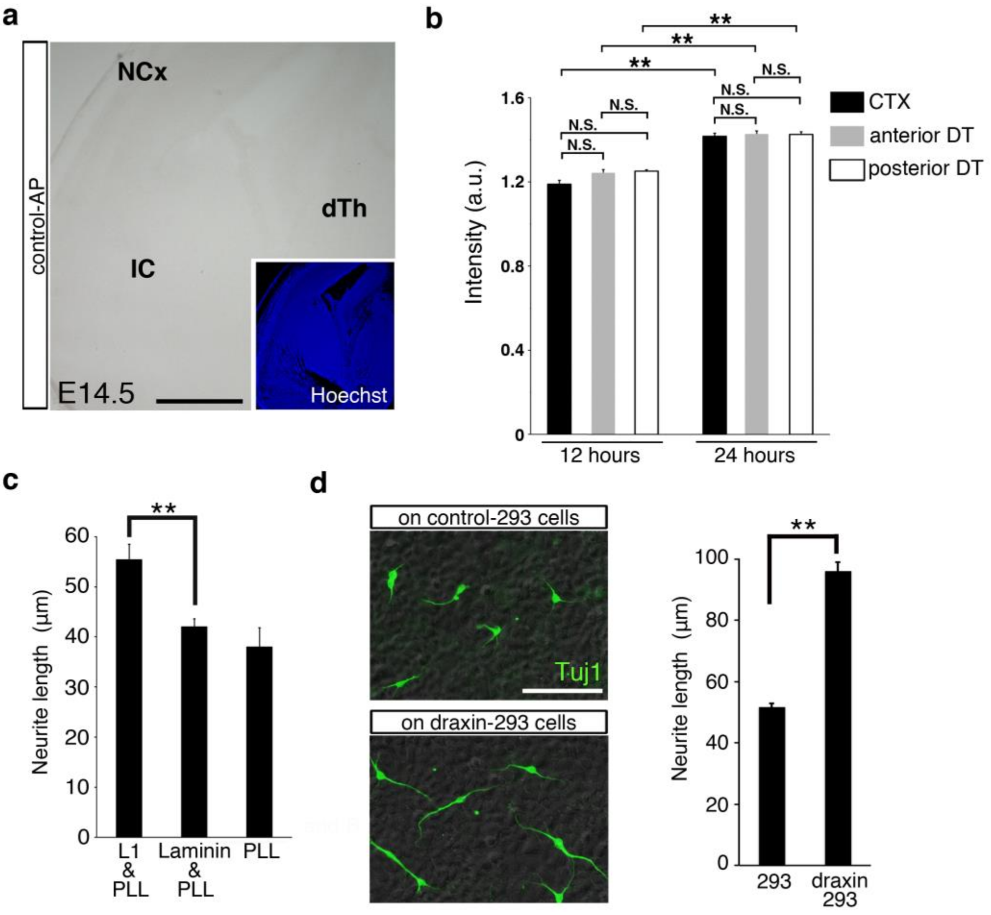

Supplementary Figure 5. Draxin binds to the neocortical and thalamic axons.

(**a**) Control-AP binding in coronal sections from E14.5 brains of wild-type mice. After an incubation in the staining buffer with BCIP and NBT, sections were stained with Hoechst 33342. No AP signal was observed in the brain sections. Scale bar, 500 µm. (**b**) Quantification of draxin-AP binding in the growth cones of dissociated neurons from the neocortex (CTX), anterior dorsal thalamus (anterior DT), and posterior dorsal thalamus (posterior DT). The AP signal intensity in these neurons was measured after 12 and 24 hours of staining. Draxin-AP binding signals in the neurons from CTX, anterior DT, or posterior DT after 24 hours were stronger than those after 12 hours. There were no significant differences in draxin-AP binding among these neurons at 12 and 24 hours. Error bars are SEM (n = 3 independent experiments). **P < 0.01 and N.S., not significant, by one-way ANOVA followed by Tukey's HSD test. (**c**) Dissociated cultures of thalamic neurons from E14.5 wild-type mice on L1/PLL-, laminin/PLL-, or PLL-coated coverslips show that L1 stimulated the neurite outgrowth. Error bars are SEM (n = 3 independent experiments). **P < 0.01 by Welch's *t*-test. (**d**) Dissociated cultures of thalamic neurons on control-293 or draxin-293 cells. Neurite outgrowth in the thalamic neurons was promoted by draxin-293 cells. Error bars are SEM (n = 4 independent experiments). **P < 0.01 by Welch's *t*-test. Scale bar, 100 µm.

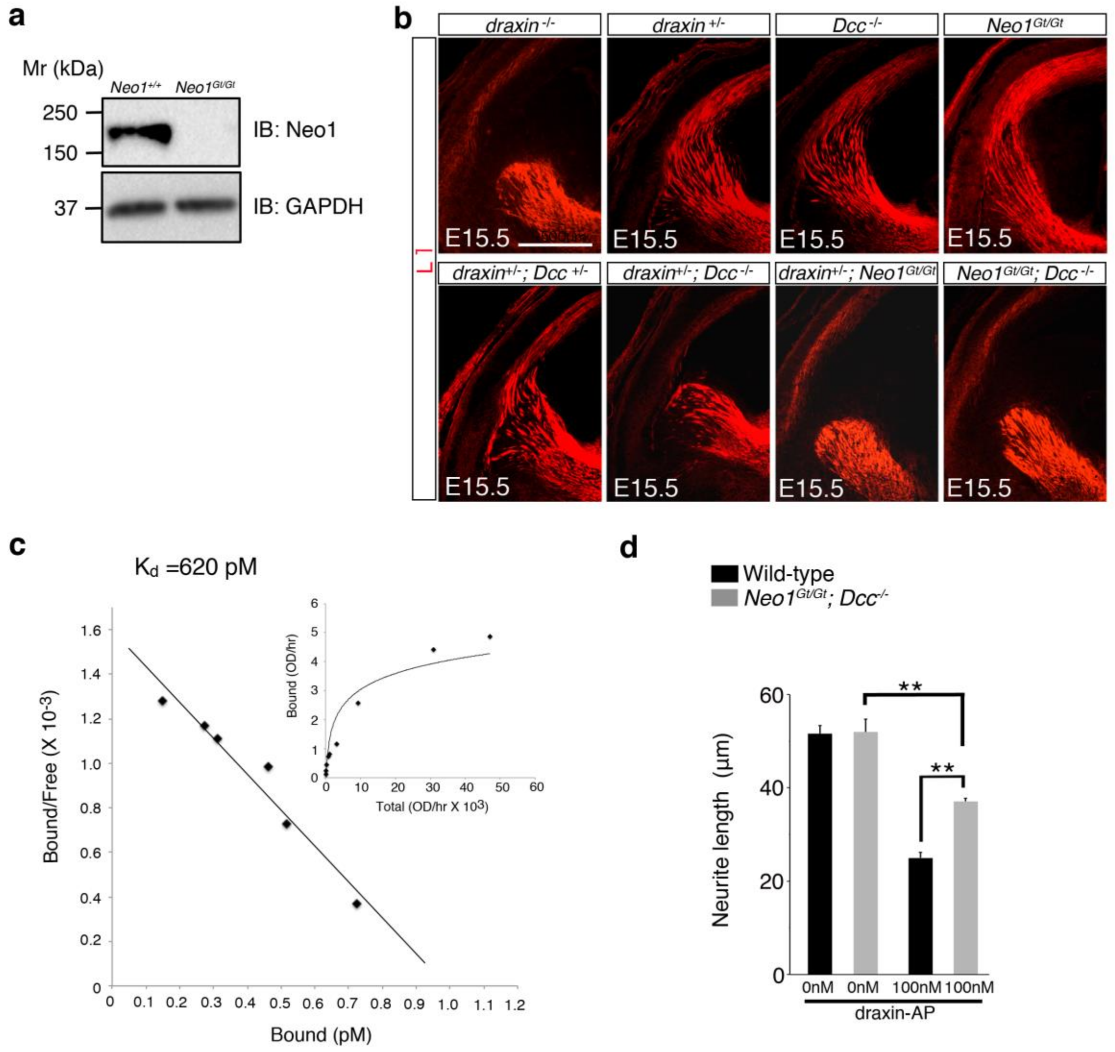

Supplementary Figure 6. DCC and Neo1 are essential components of draxin receptors.

(**a**) Western blot analysis using a Neo1 antibody revealed that the amount of Neo1 protein was dramatically reduced in the dorsal thalamus of *Neo1*$^{Gt/Gt}$ embryos at E14.5. (**b**) Coronal sections from E15.5 brains of *draxin*$^{-/-}$, *draxin*$^{+/-}$, *Dcc*$^{-/-}$, *Neo1*$^{Gt/Gt}$, *draxin*$^{+/-}$;*Dcc*$^{+/-}$, *draxin*$^{+/-}$;*Dcc*$^{-/-}$, *draxin*$^{+/-}$;*Neo1*$^{Gt/Gt}$, and *Neo1*$^{Gt/Gt}$;*Dcc*$^{-/-}$ mice stained with a L1 antibody. *draxin*$^{+/-}$;*Dcc*$^{-/-}$, *draxin*$^{+/-}$;*Neo1*$^{Gt/Gt}$ and *Neo1*$^{Gt/Gt}$;*Dcc*$^{-/-}$ mice exhibited severe defects in thalamocortical projections. Scale bar, 500 μm. (**c**) Neo1-expressing HEK293T cells were incubated with different concentrations of draxin-AP proteins, and the activities of free and bound AP were measured as described in the Methods. K_d was 620 pM. (**d**) Dissociated cultures of thalamic neurons from *Neo1*$^{Gt/Gt}$;*Dcc*$^{-/-}$ and wild-type embryos with 100 nM draxin-AP proteins. The inhibitory effect on neurite outgrowth induced by 100 nM draxin-AP was not completely abolished in the thalamic neurons from *Neo1*$^{Gt/Gt}$;*Dcc*$^{-/-}$ embryos. Error bars are SEM (n = 4 independent experiments). **$P < 0.01$, by one-way ANOVA followed by Tukey's HSD test.

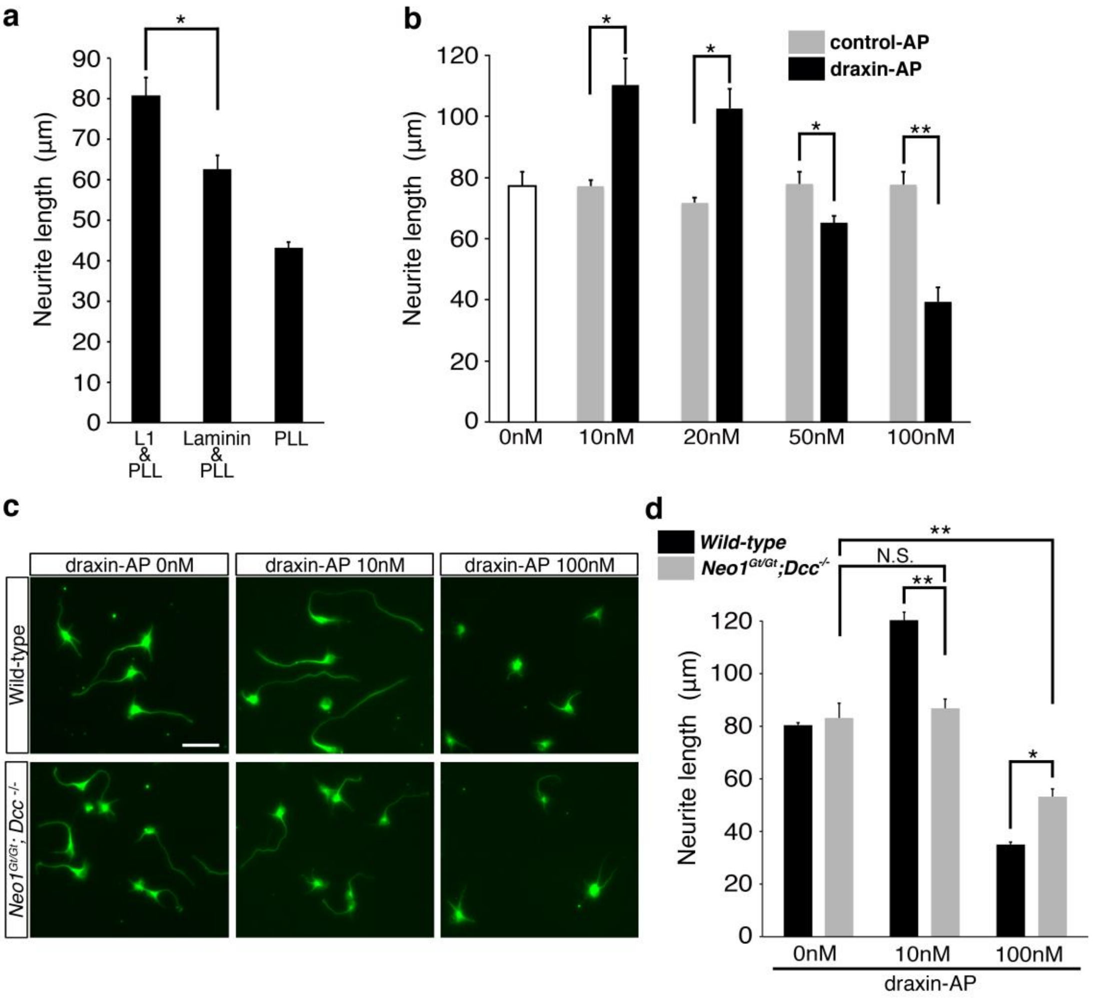

Supplementary Figure 7. Draxin regulates the neurite outgrowth of neocortical neurons. (**a**) Dissociated cultures of neocortical neurons from E14.5 wild-type mice on L1/PLL-, laminin/PLL-, or PLL-coated coverslips. Neurite outgrowth was stimulated by L1. Error bars are SEM (*n* = 3 independent experiments). *P < 0.05 by Welch's *t*-test. (**b**) Dissociated cultures of neocortical neurons with different concentrations of draxin-AP proteins showed that neurite outgrowth was promoted by draxin at lower concentrations and inhibited at higher concentrations. Error bars are SEM (*n* = 4 independent experiments). *P < 0.05 and **P < 0.01 by Welch's *t*-test. (**c**,**d**) Dissociated cultures of neocortical neurons from *Neo1*$^{Gt/Gt}$;*Dcc*$^{-/-}$ and wild-type mice with 10 and 100 nM draxin-AP proteins. The growth-promoting effect of 10 nM draxin-AP was completely abolished in neocortical neurons from *Neo1*$^{Gt/Gt}$;*Dcc*$^{-/-}$ embryos, while the inhibitory effect of 100 nM draxin-AP was partially abolished. Error bars are SEM (*n* = 3 independent experiments). *P < 0.05, **P < 0.01, and N.S., not significant by one-way ANOVA followed by Tukey's HSD test. Scale bar, 50 μm.

Supplementary Figure 4b

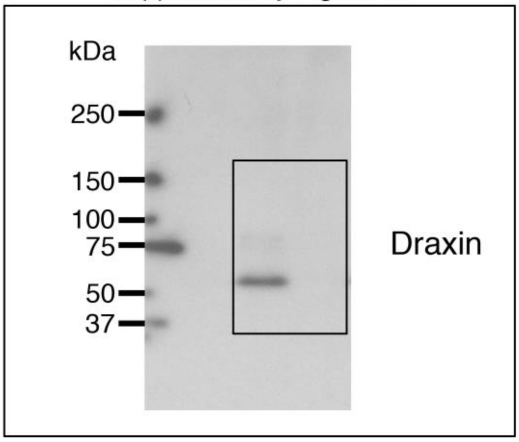

Supplementary Figure 6a

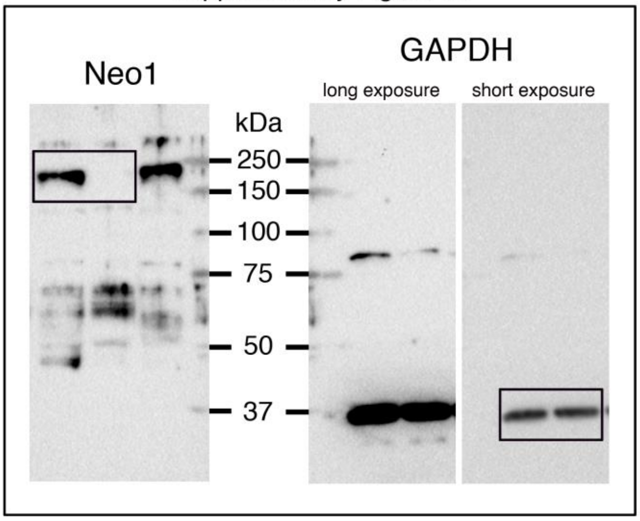

Supplementary Figure 8. Uncropped western blot images for Supplementary Figs 4b and 6a.
